# Coronin-1C and RCC2 guide mesenchymal migration by trafficking Rac1 and controlling GEF exposure

**DOI:** 10.1242/jcs.154864

**Published:** 2014-10-01

**Authors:** Rosalind C. Williamson, Christopher A. M. Cowell, Christina L. Hammond, Dylan J. M. Bergen, James A. Roper, Yi Feng, Thomas C. S. Rendall, Paul R. Race, Mark D. Bass

**Affiliations:** 1 School of Biochemistry, University of Bristol, University Walk, Bristol BS8 1TD, UK; 2 Department of Engineering, University of Bristol, University Walk, Bristol BS8 1TD, UK

**Keywords:** Coronin-1C, Coro1C, Migration, Neural crest, Rac1, RCC2

## Abstract

Sustained forward migration through a fibrillar extracellular matrix requires localization of protrusive signals. Contact with fibronectin at the tip of a cell protrusion activates Rac1, and for linear migration it is necessary to dampen Rac1 activity in off-axial positions and redistribute Rac1 from non-protrusive membrane to the leading edge. Here, we identify interactions between coronin-1C (Coro1C), RCC2 and Rac1 that focus active Rac1 to a single protrusion. Coro1C mediates release of inactive Rac1 from non-protrusive membrane and is necessary for Rac1 redistribution to a protrusive tip and fibronectin-dependent Rac1 activation. The second component, RCC2, attenuates Rac1 activation outside the protrusive tip by binding to the Rac1 switch regions and competitively inhibiting GEF action, thus preventing off-axial protrusion. Depletion of Coro1C or RCC2 by RNA interference causes loss of cell polarity that results in shunting migration in 1D or 3D culture systems. Furthermore, morpholinos against Coro1C or RCC2, or mutation of any of the binding sites in the Rac1–RCC2–Coro1C complex delays the arrival of neural crest derivatives at the correct location in developing zebrafish, demonstrating the crucial role in migration guidance *in vivo*.

## INTRODUCTION

The persistence and direction of *in vivo* cell migration is essential during development and wound healing, meaning that localization and turnover of protrusive signals are crucial. As the regulator of membrane protrusion, Rac1 is a nexus in migration signaling and is necessary for fibroblast migration along extracellular matrix (ECM) gradients, chiefly fibronectin, but not growth factor gradients (Wu et al., 2012). *In vivo*, fibroblast-specific *Rac1*^−/−^ mice suffer a wound healing defect (Liu et al., 2009) due to defects in fibroblast migration. Likewise, defective Rac1 signaling in *Danio rerio* (zebrafish) upon injection of morpholinos against the receptor responsible for Rac1 activation, syndecan-4 (Bass et al., 2007), causes developmental defects due to misregulation of Rac1 in migrating neural crest cells (Matthews et al., 2008).

One major obstacle to the use of matrix gradients as a guidance cue is that, *in vivo*, gradients along the length of a cell are very shallow (Neilson et al., 2011), and directional migration requires protrusion where the fibronectin signal is greatest, yet avoidance of additional stochastic protrusion. Classical Rac1 regulation has been found to comprise guanine-nucleotide-exchange factors (GEFs) to switch on Rac1, GTPase-activating proteins (GAPs) to switch off Rac1, and guanine-nucleotide-dissociation inhibitors (GDIs) that sequester Rac1 in the cytoplasm (Burridge and Wennerberg, 2004). However, more recently, other putative Rac1 regulators have been identified that might provide the spatial and temporal resolution required *in vivo*. One such factor is RCC2 which, despite being initially characterized for its role in mitotic spindle assembly (Mollinari et al., 2003), was recently found to be a component of fibronectin-associated adhesion complexes (Humphries et al., 2009). There is some controversy over the cytosolic role of RCC2 because it has been previously predicted to act as a Rac1 GEF, based on its similarity to RCC1 (Mollinari et al., 2003). However, RNA interference (RNAi)-mediated depletion of RCC2 enhances Rac1 activation (Humphries et al., 2009), suggesting it has an inhibitory role, and although buried residues that would result in formation of a seven-bladed propeller are conserved between RCC1 and RCC2, surface resides are poorly conserved, as is the flexible N-terminal extension (Mollinari et al., 2003). Other putative regulators include the coronin family of actin-binding proteins that regulate actin branching by inhibition of the Arp2/3 complex and stimulation of actin depolymerization by cofilin (Chan et al., 2011). Coronin-1A (Coro1A), was recently found to promote translocation of Rac1 from the cytosol to the plasma membrane (Castro-Castro et al., 2011), thereby potentially regulating Rac1 localization. By associating with the actin cytoskeleton or adhesion complexes, RCC2 and coronins are suitably localized to regulate GTPases and the key question is whether they act as non-canonical GEFs, GAPs or GDIs, second regulators of existing mechanisms or simply influence GTPases by modulating their localization.

In this manuscript, we define the molecular mechanism by which active Rac1 is focused at the tip of a protrusion. We find that the putative Rac1 regulator, RCC2 protects Rac1 from GEF-mediated activation at the plasma membrane, thereby limiting the dynamics of activation and preventing off-axial protrusion. We find that RCC2 binds coronin-1C (Coro1C), which is itself essential for redistribution and reactivation of GDP-Rac1, and therefore formation of Rac1-dependent protrusions. We find that disrupting any of these interactions causes a loss of unidirectional migration. The defect results in inefficient developmental migration of neural crest cells, so that cartilage structures are misaligned during early zebrafish development, highlighting the importance of Rac1 localization by the RCC2–Coro1C complex during development.

## RESULTS

### RCC2 as a non-canonical Rac1-sequestering molecule

**Fig. 1.**
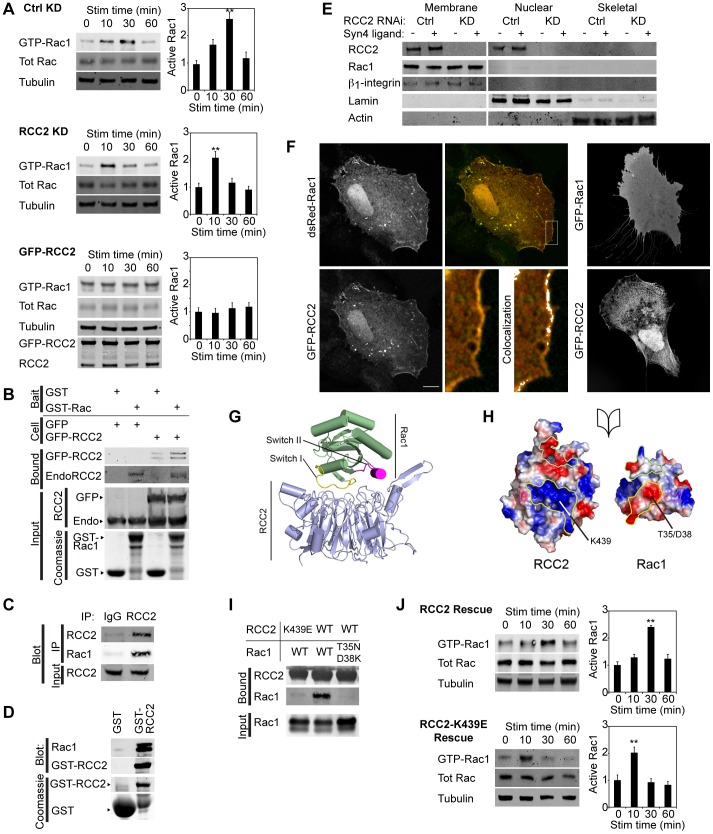
**RCC2 retards Rac1 activation at the membrane.** (A) H/0-stimulated Rac1 activation in control (Ctrl) and RCC2-knockdown (KD) MEFs (oligonucleotide no. 1, *n* = 7), and MEFs overexpressing GFP–RCC2 (*n* = 4). siRNA against RCC2 with an alternative oligonucleotide, and pairwise comparison of time points and RCC2 expression from the same experiments, is shown in supplementary material Fig. S1A–C. Stim, simulation; tot, total. (B) Pull down of endogenous RCC2 (60 kDa) and exogenous GFP–RCC2 (87 kDa) from 293T cells using GST or GDP-loaded GST–Rac1 as bait, *n* = 6. (C) Endogenous RCC2 and Rac1 co-immunoprecipitate (IP) from MEFs, *n* = 6. (D) Pull down of recombinant GDP-loaded Rac1 using GST–RCC2, *n* = 8. (E) Protein distribution between total membrane, nuclear and cytoskeletal fractions using a Qproteome kit. Comparison of control and RCC2-knockdown fibroblasts, with and without syndecan-4 engagement. *n* = 7. (F) Images of fibroblasts expressing GFP–RCC2, dsRed–Rac1 or both spread on fibronectin with serum and fixed. Colocalization tested by ImageJ. The magnified image is of the boxed area. Scale bar: 10 µm. (G) Ribbon diagram of the modeled GDP-Rac1–RCC2 complex. (H) Open-book representation of the binding interface between RCC2 and GDP-Rac1 outlining the first (yellow), second (green) and third (magenta) interaction sites. Surface charges, depicted as acidic (red) or basic (blue), complement one another at the interaction sites. (I) Interaction between GFP–RCC2 and recombinant GDP–loaded Rac1 was blocked by mutation of an interaction site in either molecule, *n* = 4. (J) H/0-stimulated Rac1 activation in RCC2-knockdown MEFs rescued with GFP–RCC2-K439E or GFP–RCC2, *n* = 4. Results are mean±s.e.m. *P<0.05, **P<0.005 (ANOVA).

Persistent migration requires polarization of signals, such as Rac1 and, although it is known that concerted engagement of fibronectin receptors, α5β1 integrin and syndecan-4, triggers Rac1 activation (Bass et al., 2007), it is less clear how the signal remains localized to a single protrusion as the composition of a 3D matrix changes. RCC2 has been identified as a negative regulator of Rac1 that associates with adhesion complexes (Humphries et al., 2009), leading us to investigate the role of a putative Rac1 inhibitor in a signaling complex traditionally linked to Rac1 activation. To synchronize receptor engagement and specifically interrogate receptor signaling, we examined how RCC2 influences the kinetics of Rac1 activation in cells spread on the α5β1-integrin-binding fragment of fibronectin, using the syndecan-4-binding fragment of fibronectin (H/0) as the trigger. Depletion of RCC2 with small interfering (si)RNA consistently accelerated Rac1 activation compared to control, such that there was a 65% increase in active Rac1 at 10 min post stimulation, a 44% decrease at 30 min, and unstimulated activity was unaffected (Fig. 1A; supplementary material Fig. S1A,B). Conversely, overexpression of GFP–RCC2 suppressed activation of Rac1 but had no effect on basal activity (Fig. 1A; supplementary material Fig. S1C). These experiments demonstrate a negative influence of RCC2 over Rac1 but suggest a block in activation, rather than inhibition per se.

Interaction between RCC2 and Rac1 was examined through binding assays. Both endogenous RCC2 and GFP–RCC2 could be isolated from 293T cells using GST–Rac1, but not GST as bait (Fig. 1B). Endogenous RCC2 and Rac1 co-immunoprecipitated (Fig. 1C), and direct interaction between bacterially expressed proteins was also detected (Fig. 1D). RCC2 was originally characterized as a component of the centromeres of metaphase chromosomes (Mollinari et al., 2003), yet the effect on Rac1 kinetics indicates that RCC2 would be expected to be associated with the membrane. RCC2 was found in both membrane and nuclear fractions of fractionated cells and the distribution was not affected by syndecan-4 engagement (Fig. 1E). Rac1 was detected in membrane and soluble fractions with RCC2 and RhoGDI (RhoGDIα, also known as ARHGDIA), respectively (supplementary material Fig. S1D). Membrane localization of RCC2 was confirmed by immunofluorescence; overexpressed GFP–RCC2 clustered and colocalized with membrane-bound DsRed–Rac1, which is normally diffusely distributed (Fig. 1F). Taken together, these experiments demonstrate that RCC2 and Rac1 localize to overlapping subcellular compartments and associate directly in fibroblasts.

To better understand the nature of the RCC2–Rac1 interaction, molecular docking was used to generate a model of the Rac1-GDP–RCC2 complex that would allow us to predict key interacting residues. A structure of RCC2 is yet to be determined, so a homology model was generated from the RCC1 structure (Renault et al., 2001) and docked with the available Rac1-GDP crystal structure (Tarricone et al., 2001), yielding a single high probability Rac1-GDP–RCC2 complex model (Fig. 1G). The ‘switch I’ loop of Rac1, including Asp38 and Thr35, sits within with a positively charged grove on the surface of RCC2 including Lys439 (yellow outline), whereas ‘switch II’ sits within a negatively charged cavity on the surface of RCC2 (green) and hydrogen bonds form at a third site (magenta) (Fig. 1H). Replacement of Lys439 of RCC2 or Thr35 and Asp38 of Rac1 completely blocked the RCC2–Rac1 interaction (Fig. 1I) and RCC2-K439E failed to rescue normal Rac1 activation in knockdown cells (Fig. 1J), confirming that RCC2 binds to the switch region of Rac1 through complementary electrostatic interactions.

**Fig. 2.**
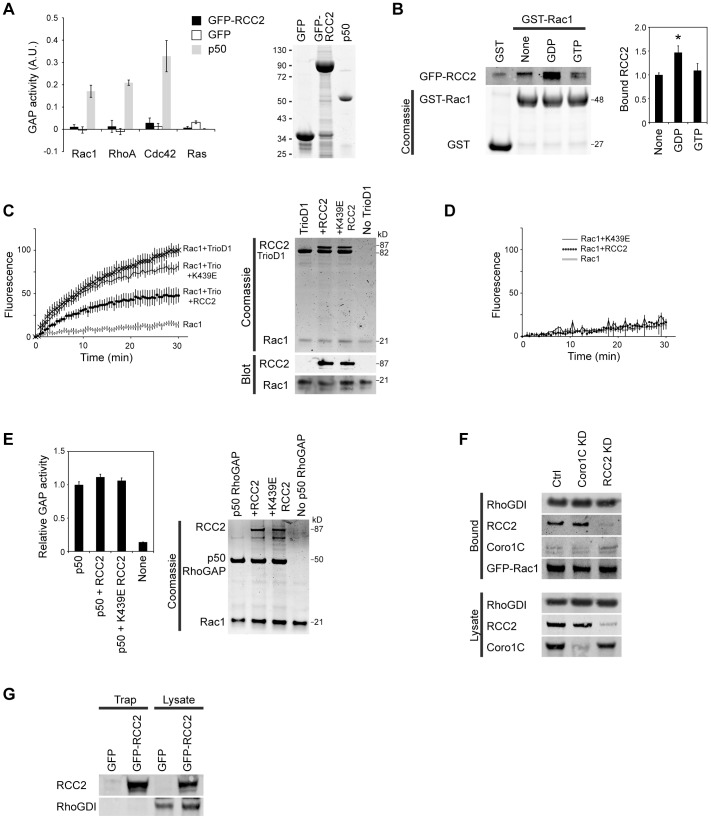
**RCC2 inhibits GEF-mediated activation of Rac1.** (A) Comparison of GTP hydrolysis by GTPases in the presence of GFP (negative control), p50RhoGAP (positive control) and GFP–RCC2, *n* = 4. The image is of a Coomassie-stained gel. (B) GFP–RCC2 from 293T lysates bound preferentially to GDP-loaded, rather than nucleotide-free or GTPγS-loaded GST–Rac1, *n* = 7. *P<0.05 (ANOVA). (C,D) GFP–RCC2, but not the RCC2-K439E mutant, inhibited TrioD1-mediated loading of mant-GTP on to Rac1 (C), but had no effect on spontaneous GTP loading (D), *n* = 12 for each. (E) GFP–RCC2 failed to inhibit p50RhoGAP-catalyzed GTP hydrolysis by Rac1, *n* = 12. (F) GFP–Rac1 was precipitated by GFP-Trap from control, RCC2-knockdown and Coro1C-knockdown MEF lysates. Co-precipitated RhoGDI, RCC2 and Coro1C were detected by western blotting, *n* = 4. (G) GFP–RCC2 was precipitated by GFP-Trap and blotted for RhoGDI and RCC2. *n* = 4. For A and C–E, tagged RCC2 and TrioD1 proteins were purified from 293T cells by GFP-Trap and relative protein loading was demonstrated by Coomassie-stained gel or western blotting. Results are mean±s.e.m.

The direct binding to the Rac1 switch region suggests that RCC2 inhibits Rac1 by either acting as a GAP, or protecting Rac1 from activation by a GEF. GFP–RCC2, purified by GFP-trap, did not show GAP activity toward Rac1, RhoA, Cdc42 or Ras (Fig. 2A). To investigate whether RCC2 might protect Rac1 from GEF action, binding of RCC2 to nucleotide-free, GDP and GTPγS-loaded Rac1 were compared. GFP–RCC2 bound to all three forms of bacterially expressed Rac1 with preference for the GDP-bound form, suggesting a sequestering role (Fig. 2B). The result was consistent with further modeling experiments that predicted that GTP-Rac1 would dock more weakly with RCC2, because the interface was confined to the switch I loop (supplementary material Fig. S1E,F). To test directly whether RCC2 prevents GEF-mediated activation of Rac1, we conducted a fluorescent guanine nucleotide exchange assay, measuring the increase in fluorescence that occurs when mant-GTP binds to Rac1. The Rac1 GEF, TrioD1 (van Rijssel et al., 2012), caused rapid loading of mant-GTP onto Rac1, compared to Rac1 alone. Addition of GFP–RCC2 to the TrioD1 reaction inhibited loading of mant-GTP, whereas addition of GFP–RCC2-K439E did not, demonstrating that RCC2 does indeed protect Rac1 from GEF-catalyzed GTP loading (Fig. 2C). RCC2 had no effect on the slow spontaneous loading of mant-GTP (Fig. 2D). RCC2 failed to inhibit GTP hydrolysis catalyzed by p50RhoGAP (also known as ARHGAP1) (Fig. 2E), which is consistent with the preference of RCC2 for the GDP-bound form of Rac1 and the accelerated Rac1 activation observed in RCC2-knockdown cells. Canonical mechanisms of Rac1 sequestration involve RhoGDI, but RCC2 knockdown had no effect on RhoGDI binding to GFP–Rac1, and RhoGDI did not co-precipitate with GFP–RCC2 (Fig. 2F,G), demonstrating that actions of RCC2 and RhoGDI are not directly linked. Taken together, these experiments demonstrate that RCC2 retards Rac1 activation by protecting Rac1 from GEF-mediated activation.

### Identification of new RCC2-binding Rac1 regulators

**Fig. 3.**
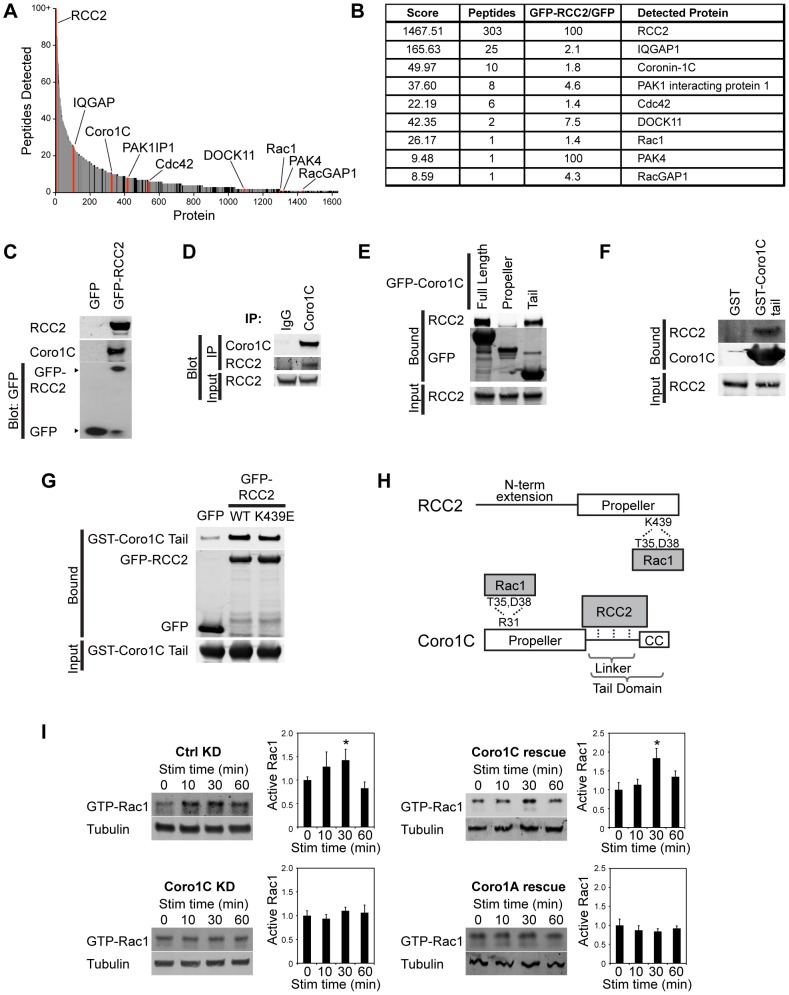
**The RCC2-binding protein Coro1C is necessary for syndecan-4-stimulated Rac1 activation.** (A) Plot of the 1636 proteins identified by SILAC mass spectrometry that associate with GFP–RCC2 better than GFP. Proteins linked to GTPase signaling are in red, nuclear and ribosomal proteins in gray, and other proteins in black. (B) A table of the proteins linked to GTPase signalling, listing score, number of peptide hits and enrichment over GFP alone. (C) Coro1C co-immunoprecipitated (IP) with GFP–RCC2 but not GFP from 293T cells in a GFP-Trap experiment, *n* = 6. (D) Endogenous RCC2 and Coro1C co-immunoprecipitated from fibroblasts, *n* = 7. (E) RCC2 co-precipitated from 293T lysates with GFP-Coro1C full length and tail domain, but not propeller domain. *n* = 5. (F) *In vitro* translated RCC2 bound to GST-Coro1C tail in a pulldown assay, *n* = 5. (G) GFP, GFP–RCC2 or GFP–RCC2-K439E beads were incubated with GST–Coro1C tail and blotted for bound tail protein. WT, wild-type. (H) Schematic of the domain structures of RCC2 and Coro1C. (I) H/0-stimulated (Stim) Rac1 activation in control (Ctrl) knockdown (KD) (*n* = 6), Coro1C-knockdown (*n* = 8), Coro1C rescue (*n* = 4) and Coro1C-knockdown and Coro1A rescue (*n* = 6), and RCC2 and Coro1C double-knockdown MEFs (*n* = 5). *P<0.05 (ANOVA). Results are mean±s.e.m. Coro1C-knockdown with an alternative oligonucleotide is shown in supplementary material Fig. S1K.

To better understand how RCC2 functions within the cell, we used stable isotope labeling with amino acids (SILAC) mass spectrometry to identify proteins that co-precipitate with GFP–RCC2, but not GFP alone. The majority of RCC2-binding partners were transcription and/or translation regulators, consistent with the nuclear localization of overexpressed RCC2. The list of proteins potentially linked to Rho-family GTPase regulation was short (Fig. 3A,B) and included Rac1 and Cdc42 as well as known regulators and effectors. Coro1C was an interesting hit, as the hematopoietic homolog, Coro1A (but not Coro1B), has been found to form a complex with RhoGDI and Rac1 in the cytosol, causing release of Rac1 from the GDI and its translocation to the plasma membrane (Castro-Castro et al., 2011), and Coro1C affects Rac1 in tumor cells by an unknown mechanism (Wang et al., 2013). We hypothesized that Coro1C might affect Rac1 localization by interaction with the RCC2–Rac1 complex. Co-precipitation of Coro1C with GFP–RCC2 or endogenous RCC2 was confirmed by western blotting (Fig. 3C,D). Coronins comprise a conserved β-propeller that includes an actin-binding site and acts as a protein scaffold, a linker region that is unique to each coronin and a coiled-coil domain that binds Arp2/3 (supplementary material Fig. S1G,H; Chan et al., 2011). Endogenous or *in vitro* transcribed RCC2 were found to bind to the linker and coiled-coil tail domain of Coro1C using GFP–Coro1C-tail or GST–Coro1C-tail as respective baits (Fig. 3E,F), and GST–Coro1C-tail still bound to GFP–RCC2-K439E, demonstrating that the interaction is not dependent on the Rac1-binding motif of RCC2 (Fig. 3G). The RCC2-binding site was mapped to the linker region of Coro1C, and further subdivision of the linker into two sections caused binding to be lost (supplementary material Fig. S1I). Taken together, these experiments demonstrate that RCC2 binds directly to the linker region of Coro1C, which is poorly conserved between coronins and suggests a specific relationship between RCC2 and the 1C isoform (Fig. 3H).

When the role of Coro1C in Rac1 regulation was tested, siRNA against Coro1C had no effect on unstimulated Rac1 activity but blocked activation of Rac1 in response to syndecan-4 engagement (Fig. 3I; supplementary material Fig. S1J,K). Rac1 activation could be rescued by re-expression of Coro1C, but not Coro1A (Fig. 3I) demonstrating that the Rac1-regulating functions of Coro1C and Coro1A are not redundant.

**Fig. 4.**
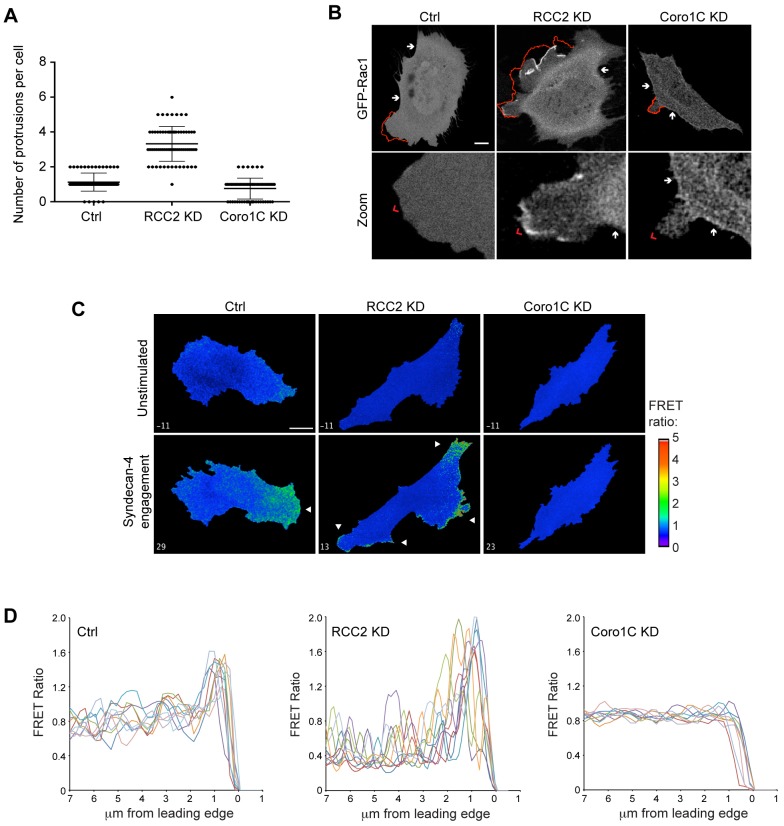
**RCC2 and Coro1C regulate Rac1 activity in protrusions.** (A,B) Control (Ctrl), RCC2 or Coro1C-knockdown (KD) MEFs expressing GFP–Rac1 were filmed on fibronectin with serum. (A) Frames were scored for number of protrusions. Results are mean±s.e.m. (B) Representative frames indicating protrusion between adjacent frames (red outline + arrowhead) and non-protrusive lateral membrane (arrow). Representative of 21 movies. (C,D) Engagement of syndecan-4 causes activation of a Raichu-Rac1 activity reporter (arrows) in a single protrusion in control, multiple protrusions in RCC2-knockdown, and no protrusions in Coro1C-knockdown MEFs. (C) Images are frames from supplementary material Movie 2. (D) FRET intensity across protruding membrane of individual cells, *n* = 12. Scale bar: 10 µm.

The contrasting effects of Coro1C and RCC2 on Rac1, the former allowing and the latter retarding activation, were reflected by the morphology and Rac1 localization of knockdown cells. Compared to the control fibroblasts, which formed a dominant protrusion on fibronectin, RCC2-knockdown cells formed very large or multiple membrane protrusions and ruffles (Fig. 4A,B; supplementary material Fig. S2A, Movie 1, red markers). In control cells, GFP–Rac1 or endogenous Rac1 were diffusely distributed but, in RCC2-knockdown cells, Rac1 accumulated in protruding membrane ruffles, consistent with accelerated activation (Fig. 4B; supplementary material Fig. S2A,B, red outlines and arrowheads, supplementary material Movie 1). By contrast, knockdown of Coro1C slightly decreased, but did not abolish, the formation of protrusions and caused Rac1 to accumulate in non-protruding membrane (from here on termed lateral membrane) (Fig. 4A,B; supplementary material Fig. S2A,B, arrows). The alterations in morphology are indicative of positive and negative effects of Coro1C and RCC2 on localized Rac1 activation, which we examined using the Raichu-Rac1 fluorescence resonance energy transfer (FRET) probe in response to syndecan-4 engagement. In control cells, Rac1 activation was polarized into a single protrusion, peaking at 30 min (Fig. 4C,D; supplementary material Movie 2). By contrast Rac1 activity peaked at 10 min in multiple protrusions in RCC2-knockdown cells, and was not activated above the basal level in Coro1C-knockdown cells, in agreement with the biochemical and confocal data.

### Coro1C facilitates redistribution of Rac1

**Fig. 5.**
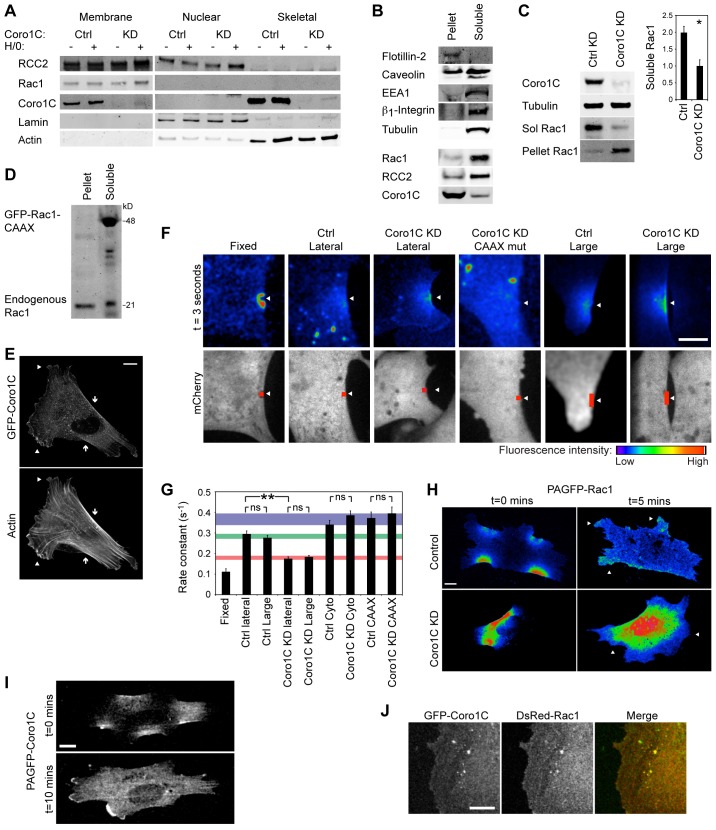
**Coro1C mediates relocalization of Rac1 from lateral to protrusive membrane.** (A) Protein distribution between total membrane, nuclear and cytoskeletal fractions using a Qproteome kit. Comparison of control (Ctrl) and Coro1C-knockdown (KD) fibroblasts, with and without syndecan-4 engagement (H/0), *n* = 4. (B) Coro1C localized to detergent-resistant (1% Nonidet P-40) membrane that includes flotillin-2 (Pellet). Rac1 and RCC2 localized to detergent-soluble lysate that includes EEA1, β1 integrin and tubulin, *n* = 8. (C) Rac1 was displaced from the detergent-soluble to insoluble fraction upon knockdown of Coro1C. *P<0.05 (Student's *t*-test), *n* = 8. (D) Mutation of the CAAX box caused GFP–Rac1 to remain in the detergent-soluble fraction of Coro1C-knockdown MEFs, *n* = 4. (E) GFP–Coro1C localized to both lateral (arrows) and ruffling (arrowheads) membrane. Representative of 100 cells. (F,G) Coro1C slows release of photoactivated (PA)GFP-tagged Rac1 from the membrane. (F) 1.5-µm or 4.5-µm sections (red boxes) of lateral membrane, identified by co-transfection with mCherry, were photoactivated at 405 nm, and dispersion of PAGFP-tagged Rac1 from the central 1.5 µm (arrows) was followed at 488 and 525 nm. Panels show sample images at 3 s post-activation, taken from supplementary material Movie 3. (G) Rate constants of fluorescent decay, comparing Coro1C-knockdown with control MEFs, using full-length or CAAX-mutant PAGFP–Rac1. **P<0.005 (by F-test); ns, not significant, *n* = 18. (H,I) In control, but not Coro1C knockdown MEFs, PAGFP-tagged Rac1 (H) and Coro1C (I) activated in lateral membrane was recruited to protruding membrane (arrowheads). Frames from supplementary material Movies 4 and 5, *n* = 15. (J) GFP–Coro1C and DsRed–Rac1 colocalize in vesicles. Results are mean±s.e.m. Scale bars: 10 µm.

The lateral mislocalization of Rac1 in Coro1C-depleted cells caused us to examine the distribution of Rac1 and Coro1C between membrane microdomains. Fractionation of cells into total membrane, nuclear and cytoskeletal fractions revealed that, as well as localizing to the cytoskeletal fraction, Coro1C could be detected in the membrane fraction, allowing proximity to RCC2 and Rac1 (Fig. 5A). Knockdown of Coro1C did not prevent association of Rac1 or RCC2 with total membrane.

Cell membranes were segregated further by separating cells into detergent-soluble [including cytosol (tubulin), early endosomes (EEA1) and soluble plasma membrane (β1 integrin)] and detergent-insoluble (including flotillin microdomains) fractions (Fig. 5B). Coro1C and RCC2 were found predominantly in the insoluble and soluble fractions, respectively, but were also detected in the alternative fractions, demonstrating that the locations do overlap. Rac1, normally found in the cytoplasm and soluble membrane of control cells, became trapped in the detergent-resistant pellet upon knockdown of Coro1C (Fig. 5C). This was the opposite of the reported effect of Coro1A knockdown, which caused increased association with RhoGDI (Castro-Castro et al., 2011). Rac1 was restored to the soluble fraction by re-expression of Coro1C but not Coro1A (supplementary material Fig. S3A). The amount of Rac1 detected in lysates prepared with 0.1% SDS was only slightly reduced, demonstrating that Coro1C regulates distribution, rather than absolute protein level of Rac1 (supplementary material Fig. S3B). Accumulation of Rac1 in the detergent-resistant pellet was due to membrane association, as a cysteine-to-serine substitution in the CAAX box caused GFP–Rac1 to remain in the soluble fraction of Coro1C-depleted cells, although the endogenous Rac1 was still detected in the detergent-resistant pellet (Fig. 5D). The shift of Rac1 to the insoluble membrane upon Coro1C knockdown, suggested that Coro1C might be responsible for release of Rac1 from detergent-resistant membrane, particularly as Coro1C localized predominantly to that fraction in control cells. The hypothesis was supported by imaging, which revealed that both GFP-tagged and endogenous Coro1C localized to the lateral membrane, in proximity to actin stress fibers, as well as ruffling membrane, and was not affected by siRNA against RCC2 (Fig. 5; supplementary material Fig. S3C,E).

Release of Rac1 from lateral membrane was measured by following dispersion of photoactivatable GFP (PAGFP)-tagged Rac1. Following photoactivation of a 1.5-µm square of lateral membrane, fluorescence quickly dispersed in control cells (*t*_½_ = 2.30 s), but was retarded by Coro1C knockdown (*t*_½_ = 3.96 s) (Fig. 5F,G; supplementary material Fig. S3F,G, Movie 3). Cysteine-to-serine substitution of the Rac1 CAAX box, to prevent membrane binding, caused dispersion of activated GFP that was as rapid as dispersion of a cytoplasmic spot (*t*_½_ = 1.86 and 2.03 s). Coro1C knockdown did not slow movement of CAAX-mutant or cytosolic Rac1 (*t*_½_ = 1.76 and 1.80 s), demonstrating that loss of Rac1 mobility in Coro1C-knockdown cells is due to membrane association. In fixed cells, where Rac1 is crosslinked to the membrane, the fluorescent signal persisted beyond the duration of the experiment. Finally, photoactivation of a 4.5-µm strip of membrane yielded similar rate constants to activation of the 1.5-µm square, demonstrating that the loss of fluorescent signal was predominantly due to inward movement of Rac1, rather than sideways diffusion within the membrane.

Interestingly, release of Rac1 from protrusive membrane was unaffected by Coro1C knockdown (supplementary material Fig. S3H), despite Coro1C localizing to both lateral and protrusive membrane. This suggested that Coro1C-mediated release of Rac1 is directional, moving Rac1 from lateral to protrusive membrane, where it is activated. Lateral PAGFP–Rac1 redistributed to protruding membrane, in control cells, but this was blocked by Coro1C knockdown (Fig. 5H; supplementary material Movie 4), demonstrating that Coro1C facilitates the redeployment of Rac1 from the edges of the cell. Furthermore, PAGFP–Coro1C itself redistributed from lateral to protrusive membrane, and Rac1 and Coro1C colocalized in small vesicles, demonstrating that they do indeed co-traffic (Fig. 5I,J; supplementary material Movie 5). Collectively, these experiments demonstrate that Coro1C redistributes Rac1 from lateral to protruding membrane and is necessary for fibronectin-induced activation, whereas the GEF inhibitor RCC2 fine-tunes the kinetics and localization of activation.

### Transfer of Rac1 between Coro1C and RCC2

**Fig. 6.**
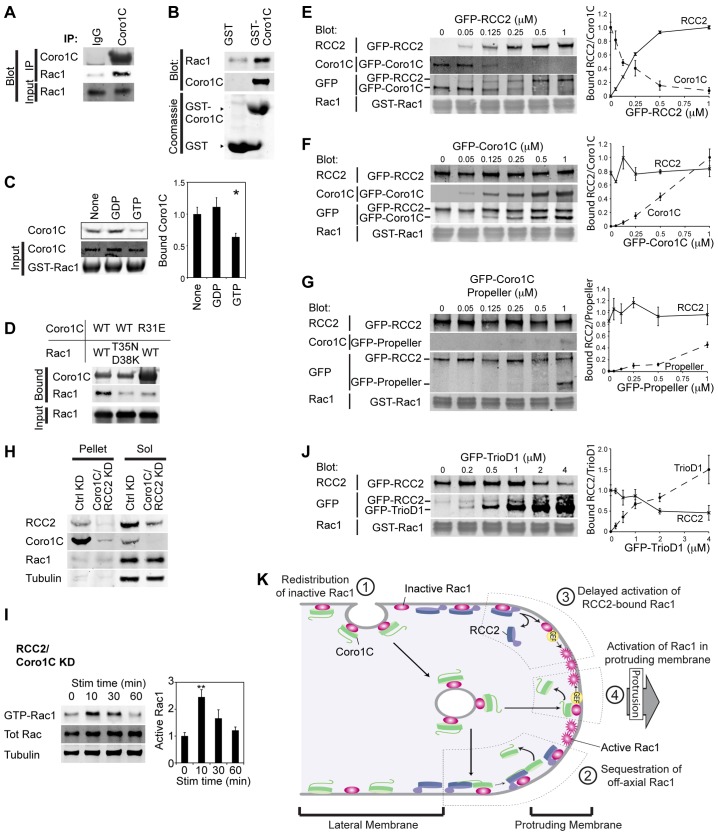
**RCC2 and Coro1C compete for an overlapping binding site on Rac1.** (A) Endogenous Coro1C and Rac1 co-immunoprecipitated (IP) from fibroblasts, *n* = 4. (B) Recombinant GDP-loaded Rac1 bound directly to GST–Coro1C, *n* = 7. (C) GFP–Coro1C from 293T lysates bound poorly to GTPγS-loaded, compared to GDP-loaded or nucleotide-free GST-Rac1, *n* = 6. (D) Interaction between GFP–Coro1C and recombinant GDP-loaded Rac1 was blocked by mutation of an interaction site of either molecule, *n* = 4. WT, wild-type. (E–G,J) GST–GDP-Rac1 beads loaded with 0.25 µM GFP–Coro1C or GFP–RCC2 in the presence of increasing concentrations of GFP–RCC2, GFP–Coro1C, GFP–Coro1C propeller or GFP–TrioD1 competitor, *n* = 4. Graphs depict average relative quantitation of bound protein in GFP blots. (H) Rac1 remained in the detergent-soluble (Sol) fraction upon knockdown (KD) of both Coro1C and RCC2, *n* = 11. (I) Syndecan-4-stimulated (Stim) Rac1 activation in RCC2- and Coro1C-double-knockdown MEFs, *n* = 6. *P<0.05, **P<0.005 (ANOVA). Results are mean±s.e.m. (K) Schematic of the roles of Coro1C and RCC2 in Rac1 retrafficking and sequestration.

The role of Coro1C in Rac1 trafficking caused us to examine possible interaction between Coro1C and Rac1. Endogenous Rac1 co-immunoprecipitated with Coro1C (Fig. 6A) and direct binding between bacterially expressed GST–Coro1C and Rac1 was also detected (Fig. 6B). A docking simulation between Coro1C and GDP-Rac1 suggested that GDP-Rac1 docks with the propeller domain of Coro1C in an arrangement similar to the RCC2–Rac1 complex. No viable solution for interaction of Coro1C with GTP-Rac1 could be found, and GFP–Coro1C bound more strongly to nucleotide-free and GDP-Rac1 than GTP-Rac1 in pulldown experiments, demonstrating that Coro1C binds poorly to active Rac1 (Fig. 6C). The docking experiment predicted that Coro1C binds to the switch regions of GDP-Rac1, including an electrostatic interaction of Arg31 of the Coro1C propeller with Thr35 and Asp38 of Rac1, and substitution of these residues perturbed the Coro1C–Rac1 interaction (Fig. 6D; supplementary material Fig. S3I). The R31E mutation had no effect on the Coro1C–RCC2 interaction or binding of Coro1C to actin (supplementary material Fig. S3J,K) and, like RCC2 knockdown, Coro1C knockdown had no effect on RhoGDI binding to Rac1 (Fig. 2F). Therefore, the binding experiments collectively demonstrate that RCC2, Coro1C and Rac1 each bind to the other components and association is not reliant on formation of a ternary complex or linked to RhoGDI.

We have demonstrated that Coro1C and RCC2 bind overlapping sites (Thr35 and Asp38) on Rac1, the former allowing release from lateral membrane, the latter retarding GEF-mediated activation. This caused us to look for binding competition between RCC2 and Coro1C (Fig. 6E–G). Coro1C could be titrated off GST–GDP-Rac1 beads by increasing concentrations of RCC2. Conversely, an equivalent concentration of Coro1C had no effect on RCC2 binding but did bind to the Rac1–RCC2 complex. We reasoned that this interaction must be due to Coro1C binding to RCC2, rather than competing for the overlapping site on Rac1 and indeed the Coro1C propeller, which includes the Rac1- but not the RCC2-binding site, did not bind except at very high concentration. Similarly, the amount of Coro1C that could be precipitated from cell lysates with GDP-Rac1 was increased by RCC2 knockdown, but Coro1C knockdown had no effect on precipitated RCC2 (supplementary material Fig. S3L,M). Taken together, these experiments demonstrate competition between RCC2 and Coro1C, where RCC2 is the preferred partner. The dominant influence of RCC2 was also demonstrated by effects on Rac1 regulation. Unlike upon Coro1C knockdown alone, cells with double RCC2 and Coro1C knockdown did not relocalize Rac1 to the detergent insoluble pellet, and instead exhibited rapid Rac1 activation and multiple Rac1-rich membrane protrusions, similar to upon RCC2 knockdown (Fig. 6H,I; supplementary material Fig. S3N,O).

Finally, we examined Rac1 release from RCC2 for activation. As RCC2, Coro1C and GEFs bind to the switch I loop, we reasoned that RCC2 and GEF would also compete for binding. At higher concentrations, TrioD1 did indeed compete RCC2 off GDP-Rac beads (Fig. 6J), which in the presence of GTP would allow nucleotide exchange (Fig. 2C), reducing affinity of Rac1 for RCC2 still further (Fig. 2B)

Collectively, these data demonstrate that Coro1C allows the release of GDP-Rac1 from lateral membrane, causing redistribution to protruding membrane where Coro1C is out-competed by RCC2 to form a sequestered pool of Rac1. Only under conditions of high GEF concentration is Rac1 activated, with the result that formation of multiple, unstable protrusions is unfavorable (Fig. 6K).

### Rac1 localization is necessary for sustained forward migration in a fibrous environment

**Fig. 7.**
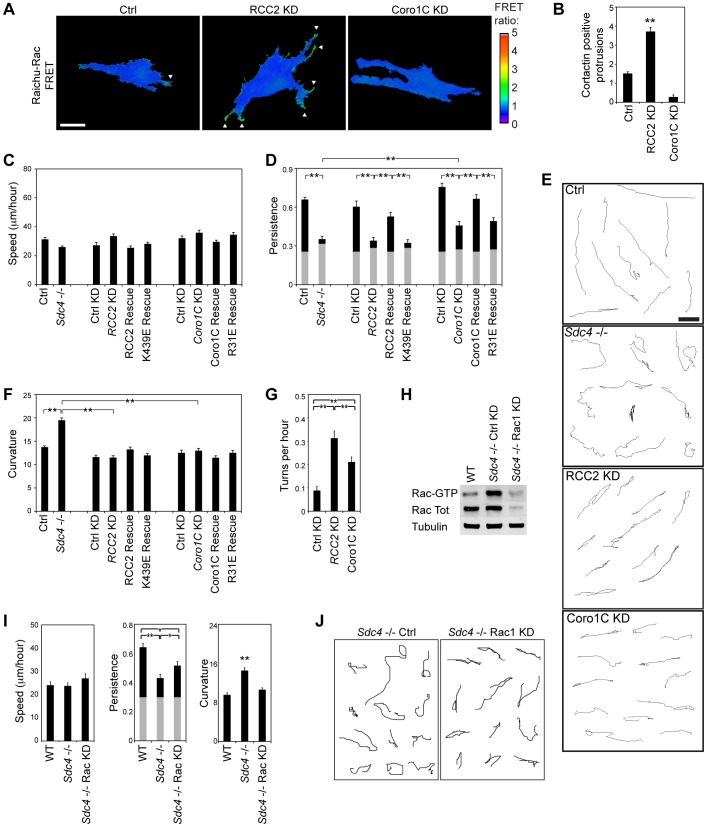
**Localization of Rac1 signals by RCC2 and Coro1C is necessary for processive migration.** (A) Distribution of active Rac1 (arrows) in control (Ctrl), RCC2-knockdown (KD) and Coro1c-knockdown MEFs embedded into CDM measured using a Raichu-Rac1 activity reporter. Images are representative of 10 experiments. Scale bar: 10 µm. (B) MEFs embedded into CDM were scored for cortactin-positive protrusions, images are shown in supplementary material Fig. S4A, *n* = 56. (C–F) 10-h migration characteristics of cell types embedded into CDM. (C) Speed (distance/time). (D) Persistence (displacement/distance), gray bars indicate the experimentally determined threshold for random migration on 2D substrate. (E) Example migration tracks. Scale bar: 100 µm. (F) Curvature (see Materials and Methods, and supplementary material Fig. S4D). (G) Frequency of migration turns on 5-µm fibronectin stripes. (H) Knockdown of total Rac1 in *sdc4*^−/−^ MEFs caused a concomitant loss of GTP-Rac1. WT, wild-type. (I,J) 10-h migration characteristics of cell types embedded into CDM. Results represent analysis of >100 cells per condition. Results are mean±s.e.m. *P<0.05, **P<0.005 (Kruskal–Wallis test).

*In vivo*, the ECM is arranged into fibers along which cells migrate by forming narrow protrusions. To examine the influence of RCC2 and Coro1C on localization of active Rac1 in a fibrous matrix, we seeded Raichu-Rac-transfected mouse embryonic fibroblasts (MEFs) into cell-derived matrix (CDM) (Bass et al., 2007). In control MEFs, active Rac1 was focused into one or two dominant protrusions (Fig. 7A,B; supplementary material Fig. S4A). RCC2 knockdown resulted in multiple narrow protrusions that resembled the formation of multiple active-Rac1 lamellae in 2D, and reflected the Rac1-sequestration defect of RCC2-depleted cells. Like the 2D FRET and biochemical analysis, Coro1C knockdown prevented activation of Rac1 in protrusions and retarded release from lateral membrane (supplementary material Fig. S4B). Therefore depletion of either RCC2 or Coro1C ablates the formation of a dominant protrusion.

We compared the migration on CDM of knockdown MEFs with wild-type, and also *Sdc4*^−/−^ MEFs that we previously reported to have a directional migration defect, due to constitutively high, mislocalized Rac1 activity (Bass et al., 2007). RCC2 or Coro1C-knockdown or *Sdc4-*knockout had negligible effect on migration speed (Fig. 7C). However control MEFs migrated persistently along matrix strands, whereas compromised expression of RCC2, Coro1C or *Sdc4-*knockout reduced directional persistence, suggesting random migration (Fig. 7D).

Inspection of the migration paths revealed that the modes of migration were very different. *Sdc4*^−/−^ MEFs moved randomly, whereas RCC2- and Coro1C-knockdown MEFs recognized the topography of the matrix but failed to commit to a dominant protrusion and shunted backwards and forwards on the fibers (Fig. 7E; supplementary material Movies 6, 7). The two types of behavior were quantified by calculating the curvature of each track (supplementary material Fig. S4C). The random migration of *Sdc4*^−/−^ resulted in a curvature value that was almost double that of control MEFs. By contrast, the curvature of RCC2- and Coro1C-depleted MEFs was similar to control MEFs, demonstrating that they followed a linear trajectory, albeit in a non-persistent fashion (Fig. 7F). The shunting migration of RCC2-depleted MEFs was consistent with the presence of active Rac1 protrusions at either end of the cell. Knockdown of RCC2 in MEFs stably expressing β1-integrin–GFP, which allowed the cell boundaries to be clearly visualized, revealed that direction changes of RCC2-depleted cells occurred when a different protrusion achieved dominance, rather than due to a tail-retraction defect (supplementary material Fig. S4D; Movies 8, 9). Notably, siRNA against Coro1C resulted in an intermediate persistence (Fig. 7D), as without a dominant protrusion Coro1C-depleted MEFs showed less commitment to a particular direction than control cells but less cause to change direction than upon RCC2 knockdown. In both RCC2- and Coro1C-knockdown MEFs, expression of wild-type protein restored persistence, but expression of Rac1-binding mutants did not (Fig. 7D; supplementary material Fig. S4E). To ensure that the shunting behavior was not due to differences in matrix organization, we analyzed migration on fibronectin stripes. Control MEFs migrated forward throughout the movie, but Coro1C-knockdown cells shunted, and RCC2-knockdown cells even more so, in agreement with the CDM data (Fig. 7G; supplementary material Movie 10).

We reasoned that if the migration phenotype of Coro1C-depleted MEFs is indeed due to constitutively low Rac1 activity, it should be possible to recapitulate the behavior by other means. To test this hypothesis, we took *Sdc4*^−/−^ MEFs that are unable to activate Rac1 in response to fibronectin but move randomly due to constitutively high Rac1 activity (Bass et al., 2007) and reduced Rac1 expression by siRNA, to a create a second cell type with constitutively low Rac1 activity. This cell type phenocopied the Coro1C-knockdown cells exactly, with low curvature and intermediate persistence values (Fig. 7H–J). Therefore we find that failure to localize Rac1 activity (RCC2-depleted), limit Rac1 activity (*Sdc4*^−/−^), or activate Rac1 in response to a fibronectin stimulus (Coro1C-depleted or *Sdc4*^−/−^ Rac1-depleted) each compromise efficient migration through a fibrillar environment.

### RCC2 and Coro1C are necessary for proper localization of neural crest derivatives in zebrafish

**Fig. 8.**
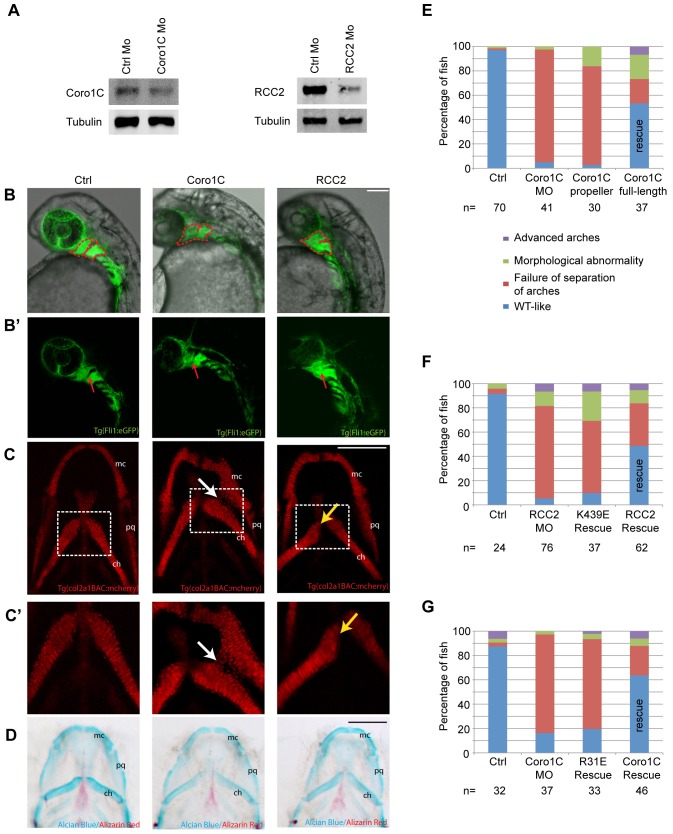
**Morpholino knockdown of Coro1C and RCC2 leads to migration defects in the developing zebrafish.** (A) Coro1C and RCC2 expression in morpholino (Mo)-injected whole zebrafish lysate at 3 dpf relative to control morpholino. (B) Lateral views at 32 hpf of Fli1:eGFP transgenic embryos, which exhibited altered migration of neural crest in the first and second pharyngeal arch elements (red dotted line), leading to mixing of the two arches (red arrows), upon injection of Coro1C (23/32 fish) or RCC2 (24/28 fish) morpholinos. (C) col2a1BAC:mCherry transgenic embryos injected with control (*n* = 49), Coro1C (*n* = 31) or RCC2 (*n* = 32) morpholinos, ventral views at 5 dpf, white arrow shows mislocalized chondrocytes between the Meckel's cartilage (mc) and ceratohyal (ch), yellow arrow shows misaligned elements. An enlargement of the boxed area is shown in C′. (D) Alcian Blue and Alizarin Red stained larvae at 5 dpf, ventral views (*n* = 28–32). (E–G) Position of Sox10:eGFP-expressing neural crest cells at 32 hpf in fish injected with control, Coro1C or RCC2 morpholinos plus rescue mRNA as appropriate. Scale bars: 100 µm.

Finally, we investigated the effect of RCC2 and Coro1C on developmental migration. RCC2 and Coro1C are widely expressed in mammals and zebrafish (Chan et al., 2011; see also, Thisse and Thisse, 2004, https://zfin.org/ZDB-PUB-040907-1). Injection of morpholinos against Coro1C or RCC2 into single-cell zebrafish embryos reduced protein expression to 52% and 17%, respectively, at 3 days post fertilization (dpf) (Fig. 8A). Morphants survived to 5 dpf with no gross anatomical defects (supplementary material Fig. S4F), demonstrating that RCC2 and Coro1C are not essential for developmental migration per se. Given that RCC2- or Coro1C-knockdown affected fibroblast migration, we examined the effect on mesenchymal lineages during development. We injected morpholinos into embryos carrying Fli1:eGFP or Sox10:eGFP transgenes, which express GFP in the migratory neural crest that populates the pharyngeal arches that will later form the cartilage elements of the ventral jaw (Lawson and Weinstein, 2002; Wada et al., 2005). Larvae were imaged at 32 h post fertilization (hpf), at which stage the crest populations of the first and second pharyngeal arches in control fish are separated by the, GFP-negative, pharyngeal pouch 1. Coro1C and RCC2 morphants demonstrated mixing of the first and second arch cells suggesting altered migratory behavior (Fig. 8B). At 5 dpf defects were also detected in the chondrocytes that are derived from the migratory crest cells. Col2a1BAC:mCherry-expressing chondrocytes mislocalized between the Meckel's cartilage and ceratohyal (derived from the first and second pharyngeal arches, respectively), such that in extreme cases the two regions merged together (Fig. 8C). The RCC2-knockdown phenotype was more subtle, resulting in misalignment of first and second arch cartilage elements such as the ceratohyal (Fig. 8C). Staining with Alcian Blue revealed cartilage maturation to be normal in morphant fish, demonstrating that reduction of RCC2 or Coro1C expression had no effect on full chondrocyte differentiation or proteoglycan synthesis (Fig. 8D). To test whether the neural crest migration defect was indeed due to the interaction between RCC2 and Coro1C, human mRNAs were co-injected with the Coro1C morpholino. Full-length Coro1C rescued separation of pharyngeal arches in 53% of fish, but a Coro1C propeller mRNA, lacking the RCC2-binding motif, failed to do so (Fig. 8E; supplementary material Fig. S4G). Furthermore, although co-injection of RCC2 or Coro1C mRNAs rescued the respective morpholino, Rac1-binding mutant mRNAs failed to do so (Fig. 8F,G). Thus, loss of any of the interactions between RCC2, Coro1C and Rac1 cause subtle but substantial alterations in mesenchymal migration *in vivo*.

## DISCUSSION

In this manuscript, we discovered a new Rac1 regulation complex comprising RCC2 and Coro1C. We find that: (1) Coro1C redistributes Rac1 from lateral to protrusive membrane; (2) RCC2 limits GEF activation of Rac1 by obscuring the switch regions and thereby prevents formation of multiple protrusions; (3) perturbation of RCC2 or Coro1C mislocalizes Rac1 and results in shunting migration; (4) suppression of RCC2 or Coro1C results in mislocalization of neural crest derivatives in developing zebrafish.

The competitive nature of the interactions of Rac1 with RCC2, Coro1C and GEFs means that local concentrations of the individual binding proteins will be important factors in determining activation rates. The bound nucleotide will also play a role. The high affinity of RCC2 for GDP-bound Rac1 will retard the initial binding of GEF, but the equilibrium will shift toward GEF binding as nucleotide exchange proceeds. This means that, in regions of low GEF activity, RCC2 would sequester Rac1, preventing stochastic activation, but in areas of high GEF activity, Rac1 will be activated efficiently, causing a cell to form a single dominant protrusion. Equally, the poor affinity of Coro1C for GTP-Rac1 means that Coro1C will effectively redistribute inactive Rac1 from lateral membrane, but not active Rac1 from protrusions (supplementary material Fig. S3H), pushing the equilibrium towards active Rac1 in a protrusion. Although the relationship of RCC2 and Coro1C could be explained in terms of relative affinity, it is probable that there will be additional regulation events. Indeed, phosphorylation of the coiled-coil domain of trimeric Coro1C prevents Arp2/3 binding and therefore organization of the actin cytoskeleton (Xavier et al., 2012). Although RCC2 binds to the linker region, rather than the coiled-coil, phosphorylation of the coiled-coil might influence RCC2 binding, either directly or by altering the localization of Coro1C. The possibility of other regulatory events affecting RCC2 or Coro1C is certainly a topic for future investigation.

It was notable that Cdc42 was also found in the RCC2 interactome and that the switch regions of all Rho family GTPases share considerable similarity. This could mean that RCC2 has broader applications than those we identify here, and it might focus the activation of other Rho family members, with the exact sites of activity depending on local GEF concentrations. It has also been predicted that *D. discoideum* coronin might bind both Rac1 and Cdc42 through a CRIB-like domain (Swaminathan et al., 2014). However, the CRIB-like domain is poorly conserved in Coro1C and the crucial histidine residue is buried. The tail domains are also poorly conserved between *D. discoideum* coronin and Coro1C, and the functions of Coro1C and Coro1A are not redundant, making it less likely that Coro1C has the broad specificity attributed to coronin in *D. discoideum*.

During mesenchymal cell migration, Rac1 must be delivered to the leading edge for activation by adhesion-receptor-associated GEFs. The two known mechanisms of Rac1 redistribution exert a negative influence on Rac1 signaling: caveolin-mediated endocytosis of polyubiquitylated Rac1 results in Rac1 degradation (del Pozo et al., 2005; Nethe et al., 2010), whereas RhoGDI extracts Rho-family GTPases for sequestration in the cytosol (Nomanbhoy et al., 1999). In this manuscript, we describe a third possibility. Coro1C releases Rac1 from lateral membrane and trafficks it to the leading edge. Because the retrafficking and the sequestering properties of the RCC2–Coro1C complex are distributed between two molecules, the sequestering phase of Rac1 redistribution can be avoided at the protruding tip, unlike in the RhoGDI pathway, which sequesters Rac1 as part of the extraction process. The benefit of the multi-component system is that Rac1 could pass through the Coro1C redistribution loop faster than it could pass through the RhoGDI sequestration loop, allowing better temporal resolution of Rac1 signaling.

In the Rac1 FRET experiments, a soluble syndecan-4 ligand caused localized Rac1 activation (supplementary material Movie 2), despite the diffuse nature of the stimulus, demonstrating the predisposition of certain regions of membrane to Rac1 activation. A role of Coro1C in maintaining high local Rac1 activity fits well with the other known functions of type I coronins. The archetypal role of coronins is prevention of Arp2/3-mediated actin branching that focuses lamellipodial protrusion. One would expect Rac1 to be retrieved from membrane where branching is inhibited and time-lapse single-molecule analysis of this process is now necessary to test the hypothesis. It was notable that the SILAC analysis that identified Coro1C as an RCC2-binding protein did not identify components of the Arp2/3 complex. The role of RCC2 in sequestering Rac1 is consistent with this finding. One would expect to find Arp2/3 at points where Rac1 is delivered for immediate reactivation, but not at points where Rac1 is sequestered. Indeed the benefit of separating the retrafficking and sequestering properties of Coro1C–RCC2 would be lost if the proteins constitutively associated.

The interactions between Rac1, RCC2 and Coro1C had pronounced effects on neural crest localization in the developing zebrafish. Likewise, syndecan-4 morphant fish exhibit a defect in neural crest migration, due to aberrant Rac1 regulation, that blocks cartilage development altogether (Matthews et al., 2008). The severity of the syndecan-4 phenotype is consistent with the migration defect of fibroblasts, because whereas *Sdc4*-knockout MEFs exhibit entirely random migration, Coro1C or RCC2-depleted MEFs can still recognize matrix fibers but lack the polarity to move processively along them, so that developmental migration is retarded, rather than completely blocked. Given the similarities *in vitro* and in zebrafish, it will be interesting to see whether Coro1C or RCC2 defects in mammals result in the defective wound healing that is the hallmark of syndecan-4-knockout mice.

## MATERIALS AND METHODS

### Cell culture

Immortalized wild-type and *Scd4*^−/−^ MEFs and human fibroblasts are as described previously (Bass et al., 2007). For RNAi, siRNA duplexes with ON TARGET^TM^ modification were transfected with Dharmafect2 reagent (Thermo Fisher Scientific). Sequences targeted the sense strand of mouse RCC2 (5′-CCAACGUGGUGGUUCGAGA-3′ or 5′-UCCAAGCGAUUCAACGUUA-3′), Coro1C (5′-CCGUUGAAUUAAUUACGUA-3′ or 5′-GUAUAAACACUCACGAGAA-3′), Rac1 (5′-AGACGGAGCUGUUGGUAAAUU-3′), human RCC2 (5′-CAAACGUGGUUGUACGAGA-3′) and Coro1C (5′-GCACAAGACUGGUCGAAUU-3′), using siGLO as control.

### Cell spreading for biochemical assays

Tissue-culture-treated plastic dishes (Corning BV) were coated with 20 µg/ml recombinant fibronectin polypeptide encompassing type III repeats 6–10 that comprises the α5β1 integrin ligand (50K) (Danen et al., 1995). To prevent *de novo* synthesis of ECM and other syndecan-4 ligands, MEFs were pretreated with 25 µg/ml cycloheximide (Sigma) for 2 h and spread for 2 h in DMEM, 4.5 g/l glucose, 25 mM HEPES, 25 µg/ml cycloheximide. Spread cells were stimulated with 10 µg/ml recombinant fibronectin polypeptide encompassing type III repeats 12–14 that comprises the syndecan-4 ligand (H/0) (Sharma et al., 1999).

### GTPase assays

Active Rac1 was precipitated from cell lysates using GST–PAK-CRIB as bait. Fluorescent Rho GEF exchange assays using 1.8 µM recombinant GTPase, 0.75 µM mant-GTP, 3 µM GFP–TrioD1 and 2 µM GFP–RCC2, and RhoGAP assays using 6.2 µM recombinant GTPase and 200 µM GTP incubated with 3.7 µM GFP–RCC2, 3.7 µM GFP or 1 µM p50RhoGAP were incubated for 20 min, before adding CytoPhos dye, according to manufacturer's instructions (Cytoskeleton).

### Cell fractionation

Spread cells were fractionated into total membrane, nuclear and cytoskeletal using a QProteome subcellular fractionation kit (QIAGEN) (Bünger et al., 2009). Detergent-resistant membrane and cytoskeletal components were isolated using 20 mM HEPES pH 7.4, 10% (v/v) glycerol, 140 mM NaCl, 1% (v/v) Nonidet P-40, 4 mM EGTA and 4 mM EDTA.

### GFP-Trap

HEK-293T cells transfected with GFP–RCC2 (Martin Humphries, University of Manchester, UK), GFP–RCC2-K439E, GFP–TrioD1 (Jaap van Buul, Sanquin, Amsterdam), GFP–Coro1C (James Bear, Howard Hughes Institute, NC), GFP–Coro1C-R31E, GFP–Coro1C propeller, GFP–Coro1C tail or GFP–C1 (Clontech) were purified by GFP-Trap (ChromoTek). Where required, proteins were eluted in glycine pH 2.5 and neutralized with Tris-HCl pH 10.4. For mass spectrometry, cells were cultured in R0K0 (GFP) or R6K4 (GFP–RCC2) SILAC media, before GFP-trap, and analyzed using a LTQ-Orbitrap Velos mass spectrometer, filtering data to satisfy false discovery rate of less than 1%.

### Binding assays

GST–Rac1 dynabeads were loaded with 6 mM GDP, 0.6 mM GTPγS or no nucleotide and used to pull down target proteins from non-clarified cell lysates or in titrations with purified proteins, where 0.25 µM GFP–Coro1C or GFP–RCC2 were bound with increasing concentrations of competitor. GST, GST–RCC2, GST–Coro1C or GST–Coro1C tail domain, immobilized on agarose beads, were incubated with soluble recombinant GDP-loaded Rac1 or *in vitro* translated RCC2, prepared using TnT quick transcription/translation kit (Promega). For co-sedimentation, 21 µM F-actin and 2 µM GFP–Coro1C proteins were co-sedimented at 150,000 ***g***.

### Western blotting

Proteins were resolved by SDS-PAGE, transferred to nitrocellulose, and analysed using the Odyssey western blotting fluorescent detection system (LI-COR Biosciences UK Ltd.). Mouse monoclonal antibodies raised against Rac1, β1-integrin, EEA1 (BD Transduction Labs), tubulin (DM1A), actin (AC-40) and Arp3 (Sigma), Coro1C (Abnova), GFP (Clontech) and polyclonal antibodies against RCC2 (Abcam), RhoGDI (Santa Cruz), caveolin (BD Transduction Labs), CCT2, flotillin-2 and Lamin A/C (Cell Signaling) were used according to the manufacturer's instructions. DyLight 680 and 800-conjugated IgGs were from Fisher.

### Homology modeling and docking studies

The RCC2 homology model was generated using the ESyPred3D server (Lambert et al., 2002) (http://www.fundp.ac.be/urdm/bioinf/esypred/), followed by model building in MODELLER and macromolecular docking with the Rac1-GDP crystal structure (Tarricone et al., 2001) (PDB 1I4D) using the ClusPro sever (Comeau et al., 2004) (http://cluspro.bu.edu/home.php). Output images were generated in PyMol (DeLano, 2002).

### Immunofluorescence

Fibroblasts transfected with pDsRed-Rac1, GFP–RCC2 or GFP–Coro1C under the control of a pMSCV promoter were spread on glass coverslips coated with 10 µg/ml fibronectin (Sigma) or CDM for 4 h in DMEM, 10% FBS, 4.5 g/l glucose, 25 mM HEPES. Where appropriate, fixed cells were stained for Rac1 (BD Transduction Labs), Coro1C (Abnova) or cortactin (Millipore), or with phalloidin, and photographed on a Leica SP5-II confocal laser scanning microscope using a 100×, NA 1.4 PlanApo objective. Maximum projection images were compiled, bandpass filtered, and analyzed using ImageJ software.

### Migration analysis

CDMs were generated as described previously by culturing confluent fibroblasts for 10 days before removing the fibroblasts by NH_4_OH lysis (Bass et al., 2007). Cells were spread on CDM or 2D fibronectin in medium with 10% serum for 4 h before capturing time-lapse images at 10-min intervals for 10 h on a Leica AS MDW microscope using a 5× NA 0.15 Fluotar or 40× NA 0.55 N PLAN objective and a Roper CCD camera. The migration paths of all non-dividing, non-clustered cells were tracked using ImageJ software.

Persistence was calculated by dividing linear displacement of a cell over 10 h by the total distance migrated. The total absolute curvature of a track curvature, *C*, is defined by:
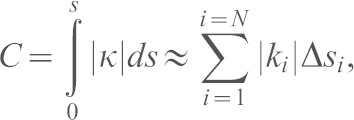
where *k_i_* is the curvature at the mid-point of a straight track segment *i*, Δ*s_i_* is the length of that segment and the curvature *κ* for a parametric curve 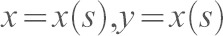 is given by:
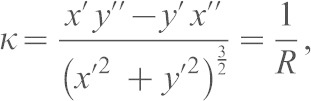
with
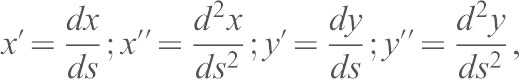
where *s* is the arc length along the track (Cui et al., 2009). Interpretation of *C*, illustrated in supplementary material Fig. S4C, is straightforward. Regions of high curvature correspond to regions with a small radius of curvature, where the track is tightly curved and changing direction rapidly.

For migration on 5-µm fibronectin stripes (Cytoo), cells were seeded in medium with 2% serum and allowed to spread for 2 h before capturing time-lapse images at 10-min intervals for 6 h on a Leica AS MDW microscope using a 10× NA 0.3 objective and a Roper CCD camera. Cells were scored for number of direction changes during the movie.

### FRET analysis of Rac activity

Fibroblasts transfected with the Raichu-Rac probe (Itoh et al., 2002) were filmed on 50K-coated MATTEK dishes for 10 min, before stimulation with H/0 for 40 min. Images were acquired on a Leica DM IRBE inverted microscope using a 63× NA 1.32 objective, Sutter DG5 light source and Photometrics Coolsnap HQ2 camera, capturing images through CFP and YFP filters upon excitation through the CFP channel every 2 min. FRET ratios were calculated as described previously (Machacek et al., 2009) using ImageJ software. Briefly, aligned CFP and YFP images were corrected for uneven illumination, photobleaching and background was subtracted. A binary mask was used to define the borders of the cell, and the YFP image was divided by the CFP image to yield a ratio image reflecting the distribution of Rac1 activity across the cell. The same result was obtained when the order of acquisition of CFP and YFP images was reversed or when fixed cells were analyzed, eliminating motion artifacts.

### Photoactivatable-GFP–Rac1

Control or Coro1C-knockdown MEFs were co-transfected with pmCherry and PAGFP-tagged Rac1, Rac1-CAAX (cysteine to serine mutation) or Coro1C, and spread on fibronectin or CDM. A 1.5-µm×1.5-µm or 1.5-µm×4.5-µm box on the lateral membrane was photoactivated at 405 nm and then GFP fluorescence at 488 nm was recorded on an Ultraview Spinning Disk confocal microscope (Perkin Elmer) using a 63×, NA 1.3 PlanApo objective. For diffusion experiments, images were captured at 2 images per second for 5 s prior to, and 15 s after, photoactivation. For re-trafficking experiments, PAGFP was photoactivated at three or four separate 1.5-µm×4.5-µm boxes and then images captured at one image per min for 10 min. For diffusion experiments, pixel intensity within a 1.5-µm×1.5-µm box at the center of the activated area was measured and one-phase decay curves fitted using GraphPad Prism. Significant difference between decay curves was tested using F-tests.

### Zebrafish husbandry and analysis

All experiments were carried out according to UK Home Office regulations. Lines used were Tg(col2a1BAC:mCherry) (Hammond and Schulte-Merker, 2009), Tg(Fli1:eGFP)^y1^ (Lawson and Weinstein, 2002) and Sox10:eGFP (Wada et al., 2005). Morpholinos (Gene Tools) against coronin-1ca (ENSEMBL gene ID: ENSDARG00000035598) (translation blocking, 5′-TCGTACAACCCGTTTGAACATATCT-3′, 3.5 nM) and RCC2 (ENSEMBL gene ID: ENSDARG00000011510) (splice blocking, 5′-ATACAAGAAGCATCCTTACAATCTT-3′, 1 ng) were injected at the one-cell stage. For rescue experiments, 200 pg human mRNA, prepared using a mMessage mMachine kit (Ambion), were co-injected with the morpholino. Images were captured on a Leica SP5-II confocal laser-scanning microscope. Alcian Blue and Alizarin Red staining is as previously described (Walker and Kimmel, 2007).

## Supplementary Material

Supplementary Material
