# Supplementary material for: Coronin-1C and RCC2 guide mesenchymal migration by trafficking Rac1 and controlling GEF exposure
Source: J Cell Sci. 2014 Oct 1;127(19):4292–307. doi: 10.1242/jcs.154864 (PMC4179493; doi:10.1242/jcs.154864)
Supplement: Supplementary Material [file supp_127_19_4292__index.html]

Supplementary Material 

# Coronin-1C and RCC2 guide mesenchymal migration by trafficking Rac1 and controlling GEF exposure

## JCS154864 Supplementary Material

**Files in this Data Supplement:**

- **Supplementary Material**
