# Supplementary material for: Coronin-1C and RCC2 guide mesenchymal migration by trafficking Rac1 and controlling GEF exposure
Source: J Cell Sci. 2014 Oct 1;127(19):4292–307. doi: 10.1242/jcs.154864 (PMC4179493; doi:10.1242/jcs.154864)
Supplement: Supplementary Material [file supp_jcs.154864_JCS154864.pdf]

Supplemental Information

A coronin-1C/RCC2 complex guides mesenchymal migration by trafficking Rac1 and controlling GEF exposure

Rosalind C. Williamson, Christopher A. M. Cowell, Christina L. Hammond, Dylan Bergen, James Roper, Yi Feng, Thomas C. S. Rendall, Paul R. Race and Mark D. Bass

Fig. S1. RCC2 and Coro1C are Rac1 regulators

(A) Syndecan-4-stimulated Rac1 activation in RCC2 knockdown MEFs. Experiment uses an alternative oligo sequence to that shown in Fig. 1A. n=3.

(B) Pairwise comparison of syndecan-4-stimulated Rac1 activation at 0 and 10 minutes stimulation, in control and RCC2-knockdown MEFs (oligo #1), the complete timecourses are shown in Fig. 1A. n =7.

(C) Comparison of Rac1 activation in unstimulated control MEFs and MEFs overexpressing GFP-RCC2. n = 4.

(D) Distribution of Rac1, RhoGDI and RCC2 between soluble (CCT2) and total membrane (β_1-integrin) fractions using a Qproteome kit. n=7.

(E) Scores for *in silico* docking experiments between RCC2/Coro1C and GDP/GTP-loaded Rac1.

(F) Binding interface between RCC2 and GTP-Rac1 from *in silico* docking experiments, demonstrating that interactions are confined to the Switch 1 loop of GTP-Rac1.

(G-H) Coro1C can be divided into functional subdomains. (E) Arp3 can be pulled-down from lysate with GST-Coro1C-tail. (F) GFP-Coro1C full length and propeller domain cosediment with filamentous actin. n=4.

(I) RCC2 coprecipitated from 293T lysates with GFP-Coro1C linker region (residues 351-435), but not the coiled-coil domain (residues 436-474). Further subdivision of the linker into N- and C-terminal parts (residues 351-397 and 393-435 respectively) also caused loss of binding. n=4.

(J) Knockdown of Coro1C had no effect on Rac1 activity in unstimulated MEFs. n=8.

(K) Syndecan-4-stimulated Rac1 activation in Coro1C knockdown MEFs. Experiment uses an alternative oligo sequence to that shown in Fig. 3I. n=4.

Error bars indicate s.e.m.

Fig. S2. Coro1C and RCC2 regulate Rac1 localization

(A) Control, RCC2-knockdown, Coro1C-knockdown, RCC2/Rac1-knockdown, or Coro1C/Rac1-knockdown MEFs were spread on fibronectin in the presence of serum. Cells were fixed and stained with phalloidin and immunostained for endogenous Rac1. Rac1 accumulated in actin-rich lamella (arrowheads) of RCC2-knockdown MEFs and lateral membrane (arrows) of Coro1C-knockdown MEFs. Images are representative of 100 cells on 4 separate occasions.

(B) GFP-Rac1 accumulation in protrusions of RCC2-knockdown MEFs and along the sides of Coro1C-knockdown MEFs spread on fibronectin in the presence of serum. Intensity profiles were measured across protrusions (red) and lateral membrane (green). 13 profiles, randomly selected from 100 cells from 4 separate experiments are displayed.

Fig. S3. Competition between Coro1C and RCC2 cause Rac1 redistribution

(A) Rac1 was restored to the detergent-soluble fraction of Coro1C-knockdown MEFs by exogenous expression of Coro1C, but not Coro1A. n=4.

(B) Knockdown of Coro1C has no affect on total Rac1 protein levels, lysates prepared with 0.1% SDS to ensure extraction of Rac1 from all membrane fractions. n=4.

(C) Immunofluorescent staining of endogenous Coro1C demonstrating localization to both actin-rich ruffles (arrowheads) and lateral membrane (arrows). Staining is ablated by knockdown of Coro1C. Images are representative of 100 cells on 3 separate occasions.

(D) Fibroblast expressing GFP-Coro1C following RCC2 knockdown, spread on fibronectin with serum and fixed. Image representative of 50 cells on 2 separate occasions.

(E) Protein distribution between soluble, total membrane, nuclear and cytoskeletal fractions. Coro1C is still found in soluble, total membrane and cytoskeletal fractions upon RCC2-knockdown. n=7.

(F) Fluorescent decay curves compare redistribution of photoactivated GFP-tagged Rac1 from lateral membrane between control and Coro1C-knockdown MEFs.

(G) Rate constants of fluorescent decay, comparing Coro1C-knockdown with control MEFs, using alternative oligos to those presented in Fig. 5G. n=18.

(H) Rate constants of fluorescent decay, comparing dispersion of PAGFP-Rac1 from protrusive membrane Coro1C-knockdown with control MEFs, including dispersion from lateral membrane of control cells for comparison. n=18.
(I) Binding interface between Coro1C and GDP-Rac1 from *in silico* docking experiments, demonstrating that interactions include Thr35 and Asp38, similar to the RCC2/GDP-Rac1 complex.
(J) RCC2 coprecipitates with GFP-Coro1C and GFP-Coro1C-R31E with similar efficiency, using a GFP-Trap from 293T cells. n=3
(K) Wild type and R31E Coro1C cosediment with freshly polymerized filamentous actin at 150,000xg, whereas the previously charactized actin-binding mutant of Coro1C, R28D/2xKE, does not.
(L-M) Competition between RCC2 and Coro1C for binding to Rac1. Pull down assays from lysates of control, RCC2-knockdown or Coro1C-knockdown cells using GST or GDP-loaded GST Rac1 as bait. n=6
(N-O) Knockdown of both RCC2 and Coro1C results in a morphology that resembles RCC2 knockdown. Cells form multiple lamellae (arrowheads) on fibronectin, to which both immunostained endogenous Rac1 and GFP-Rac1 localize. Images are reproduced, in part, in Fig. S2B. n=70.
Error bars indicate s.e.m. Significance was tested by T-test, ** $p<0.005$. Bar = 10 µm.

Fig. S4. RCC2 and Coro1C regulate migration

(A) MEFs spread on CDM with serum, fixed and stained for cortactin and fibronectin. Images representative of 100 cells on 2 separate occasions. Bar = 10 µm.
(B) Fluorescent decay curves compare redistribution of photoactivated GFP-tagged Rac1 from lateral membrane between control and Coro1C-knockdown MEFs plated on CDM. n=18.
(C) Schematic of how the angle of each step of a migration path was assessed to calculate curvature of path.
(D) Cell outlines illustrating a migration sequence, red>yellow>green>cyan>blue to show that control cells are processive, while RCC2-depleted cells shunt. Individual frames derived from Movies S8-9.
(E) Lysates of control, RCC2, and Coro1C knockdown and rescued MEFs, used in Fig. 7C-F) were blotted for RCC2 to confirm knockdown and rescue.

(F) Lateral views of control, Coro1C or RCC2 morphants, at 4 dpf.

(G) Confocal stacks at 32 hours post fertilisation of the neural crest reporter line: Tg(-4.9sox10:EGFP)ba2 (Wada et al, 2005). Images are all of left-facing zebrafish heads, anterior to top. 1=neural crest stream from which 1st arch skeletal elements will be derived, 2= neural crest stream from which 2nd arch skeletal elements are derived, 1/2 indicates failed separation of these two streams. Fish were staged as 32 hpf by reference to migration of sox10:GFP labelled pigment precursors in the trunk. Ctrl are control morpholino injected (n=70), Mo only= Coro1C morpholino injected only (at 2ng per embryo)(n=41), Mo+mut RNA = Coro1C morpholino (2ng) in addition to 200 pg truncated Coro1C RNA (n=37), Mo+wt RNA= Coro1C morpholino (2ng) in addition to 200 pg full-length Coro1C RNA (n=30). All embryos were injected directly into the first cell at the 1-cell stage of development.

Error bars indicate s.e.m. Significance was tested by T-test, ** $p<0.005$.

Movie 1. RCC2 and Coro1C regulate Rac1 localization and membrane protrusion. Control, RCC2 or Coro1C knockdown MEFs were transfected with GFP-Rac1 and filmed on a confocal microscope for 3.5 hours at 1 frame every 3 minutes. Red dots indicate protrusion between consecutive frames. Movie frames reproduced in Fig. 4B.

Movie 2. Localization of Rac1 activation is perturbed in RCC2 or Coro1C knockdown MEFs. Rac1 activity distribution was detected using a Raichu-Rac FRET probe in cells spread on 50K before addition of H/0 (white flash). A non-activatable mutant probe (Y40C) was used as a control to confirm that changes in FRET signal are caused by changes in Rac1 activity, not relocalization. Movie captured at 1 frame every 2 minutes, for 11 minutes prior to, and up to 49 minutes after stimulation. Images are false-colored for FRET intensity. Movie frames reproduced in Fig. 4C.

Movie 3. Release of photoactivated GFP-tagged Rac1 from the membrane is delayed in the absence of Coro1C expression. PAGFP was photoactivated in a 1.5x1.5 μm or 1.5x4.5 μm box at the lateral edge of cells spread on fibronectin and release of Rac1 followed by decay of GFP fluorescence. Fixed cells (no diffusion), control MEFs (large and small boxes), Coro1C-depleted MEFs (large and small

boxes) and Coro1C-depleted MEFs using a -CAAX mutant Rac1 (no association of PAGFP-Rac1 with membrane) were analyzed. Images are false-colored for fluorescence intensity. Images were captured at 2 images per second for 5 seconds prior to, and 15 seconds after photoactivation, and displayed at 2 frames per second. Movie frames reproduced in Fig. 5F.

Movie 4. Retrafficking of Rac1 from lateral to protrusive membrane is reliant on Coro1C. PAGFP-Rac1 was photoactivated in boxes at the lateral edge of control or Coro1C KD cells spread on fibronectin, and arrival at protrusive membrane recorded. Images are false-colored for fluorescence intensity. Images were captured at 1 image per minute for 10 minutes following photoactivation and displayed a 1 frame per second. Movie frames reproduced in Fig. 5H.

Movie 5. Retrafficking of Coro1C from lateral to protrusive membrane. PAGFP-Coro1C was photoactivated by 3 pulses within 1 minute at boxes on the lateral membrane of cells spread on fibronectin, and arrival at protrusive membrane recorded over 10 minutes at 1 image per minute for 10 minutes following photoactivation. Movie frames reproduced in Fig. 5I.

Movie 6. RCC2 and Coro1C expression are necessary for processive migration. Control, RCC2 and Coro1C knockdown and *Sdc4* -/- MEFs migrating through a cell-derived matrix. Movie captured with a 5x lens at 1 image every 10 minutes for 10 hours, displayed at 5 frames per second, bar = 100 μm.

Movie 7. RCC2 and Coro1C expression are necessary for processive migration. Control, RCC2 and Coro1C knockdown MEFs migrating through a cell-derived matrix. Movie captured with a 40x lens at 1 image every 10 minutes for 10 hours, displayed at 5 frames per second, bar = 10 μm.

Movie 8. Shunting migration of RCC2 knockdown MEFs is not due to a tail retraction defect. β_1-integrin-GFP-expressing MEFs transfected with control oligos were filmed migrating through a cell-derived matrix to allow the rearmost attachment point to be seen. Movie captured at 1 frame every 10 minutes, displayed at 6 frames per second.

Movie 9. Shunting migration of RCC2 knockdown MEFs is not due to a tail retraction defect. β_1-integrin-GFP-expressing MEFs transfected with RCC2-targeted antisense oligo were filmed migrating through a cell-derived matrix to allow the rearmost attachment point to be seen. Movie captured at 1 frame every 10 minutes, displayed at 6 frames per second.

Movie 10. RCC2 and Coro1C expression are necessary for processive migration. Control, RCC2 and Coro1C knockdown MEFs migrating along 5-μm fibronectin stripes. Movie captured at 1 image every 10 minutes for 6.5 hours, displayed at 3 frames per second, bar = 50 μm.

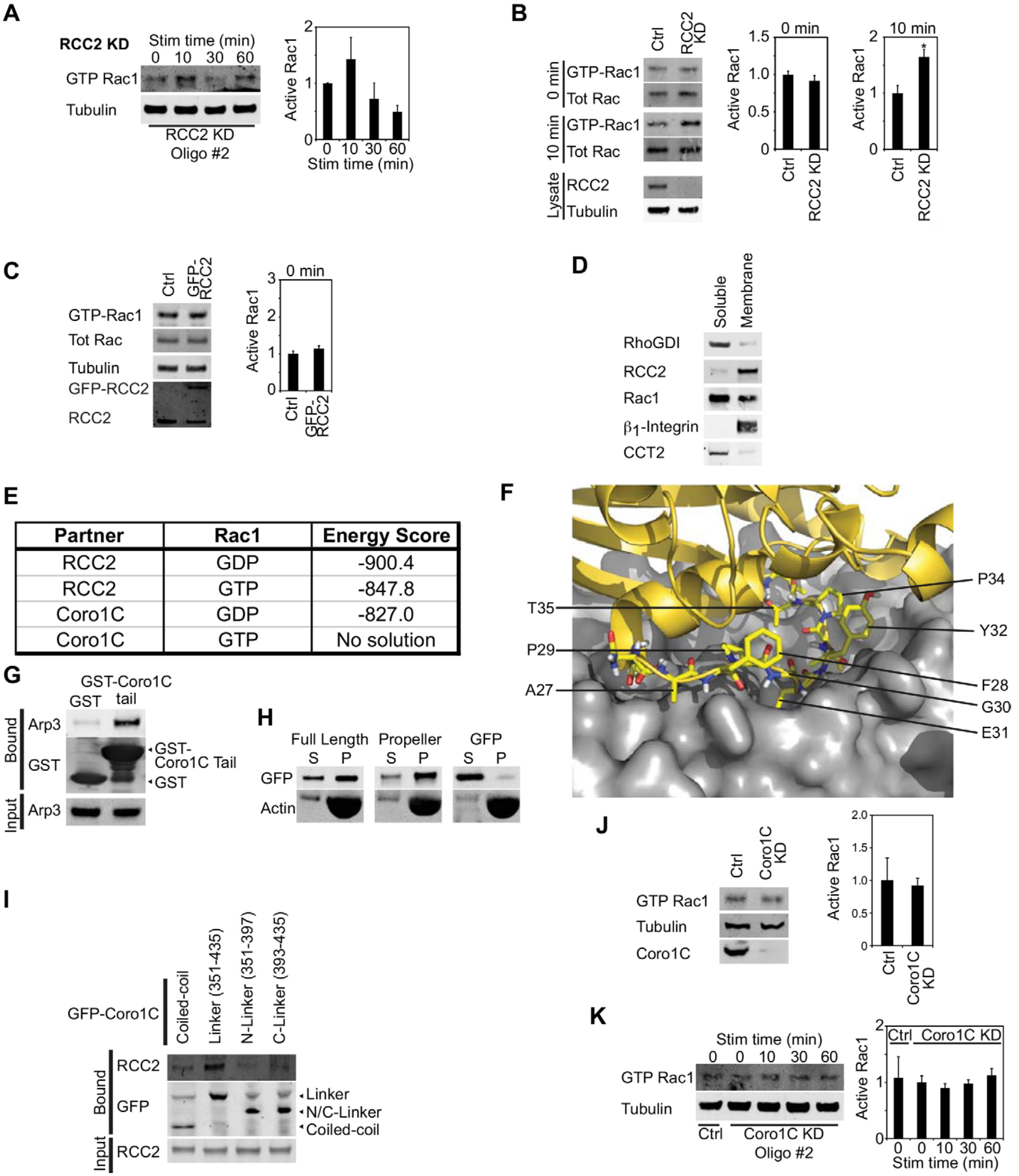

Partner	Rac1	Energy Score
RCC2	GDP	-900.4
RCC2	GTP	-847.8
Coro1C	GDP	-827.0
Coro1C	GTP	No solution

Figure S1.

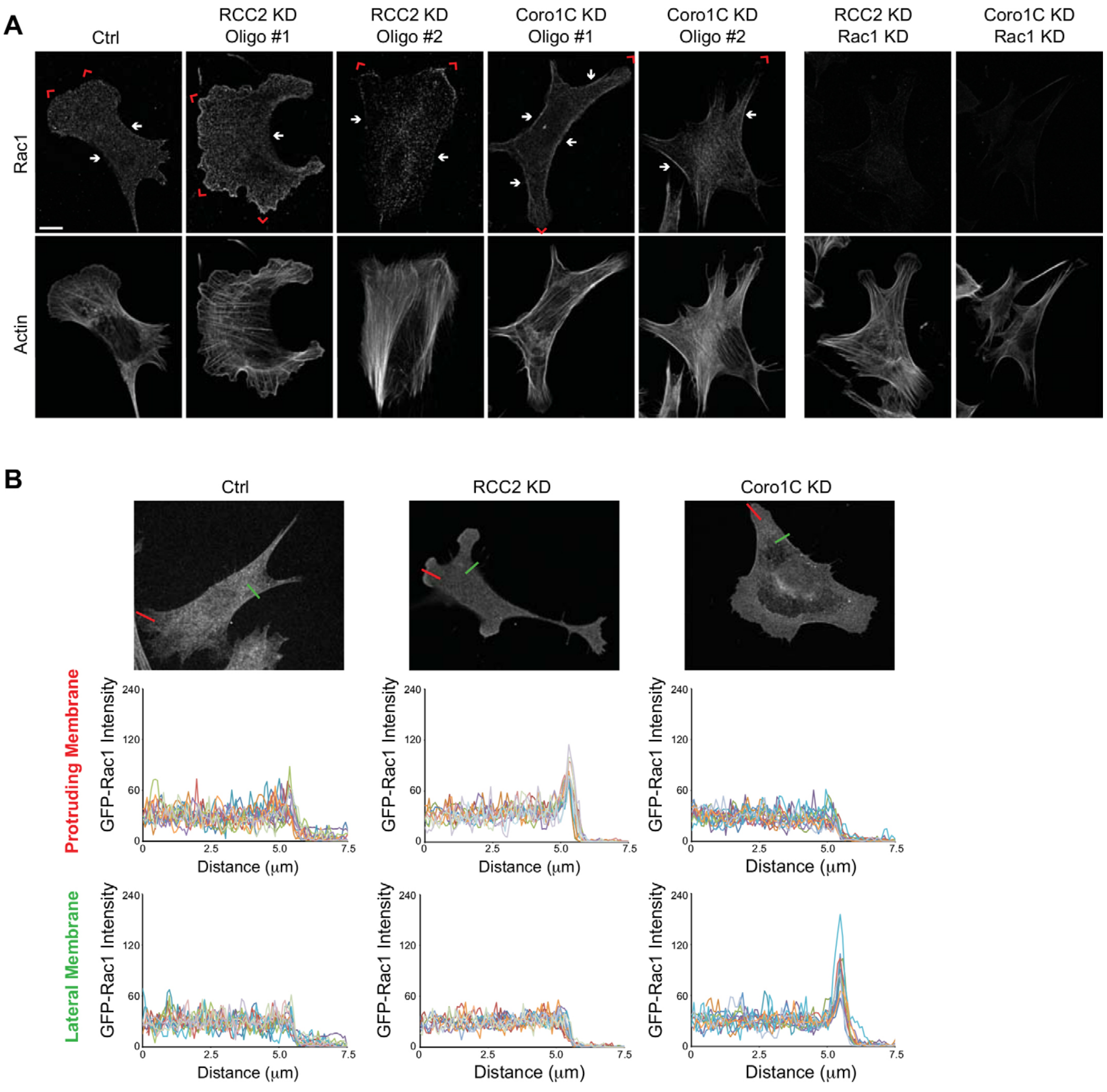
A
Ctrl
RCC2 KD Oligo #1
RCC2 KD Oligo #2
Coro1C KD Oligo #1
Coro1C KD Oligo #2
RCC2 KD Rac1 KD
Coro1C KD Rac1 KD
Rac1
Actin
B
Ctrl
RCC2 KD
Coro1C KD
Protruding Membrane
Lateral Membrane
GFP-Rac1 Intensity
Distance (µm)
0
60
120
240
2.5
5.0
7.5

Figure S2.

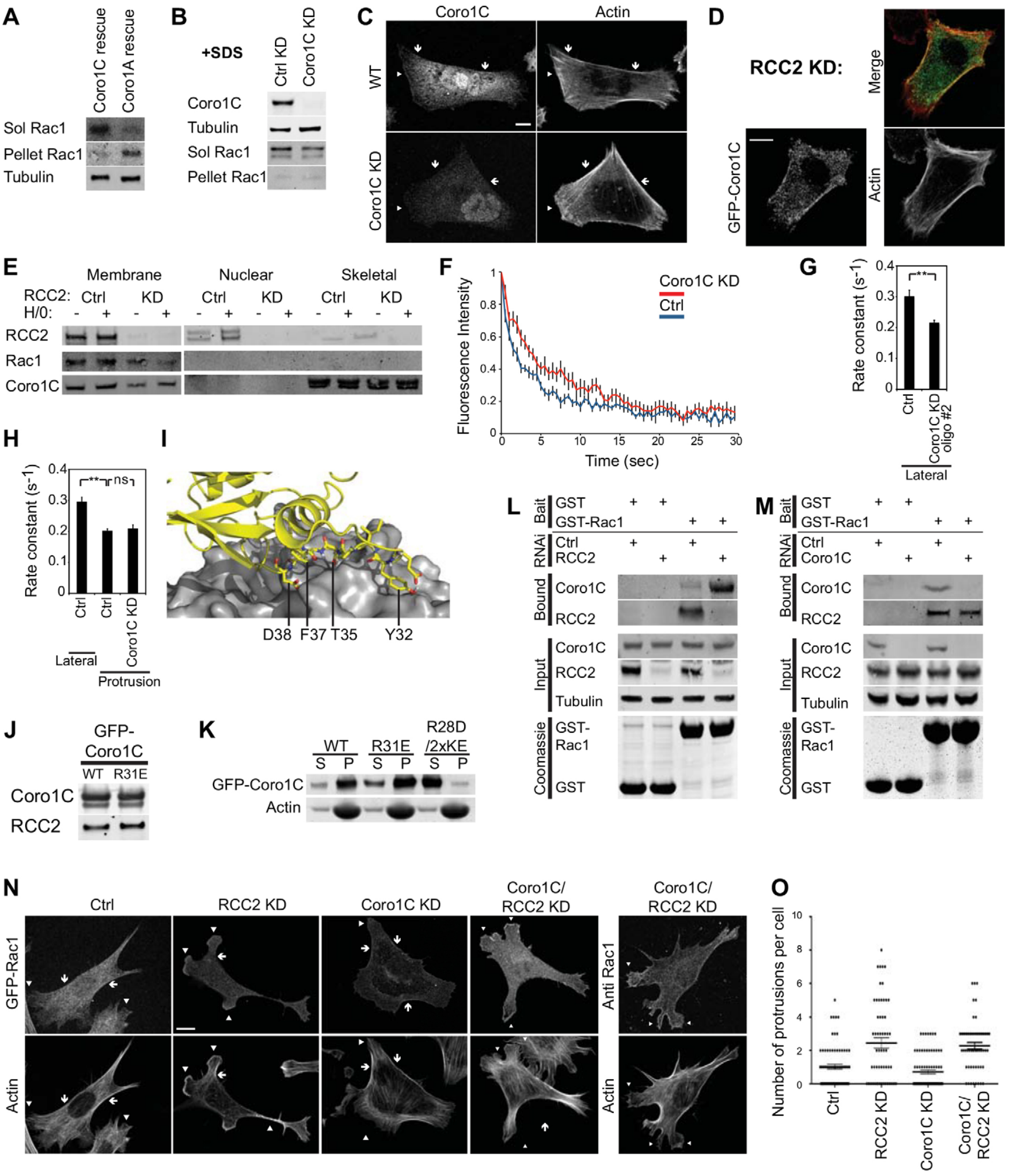
A
Coro1C rescue
Coro1A rescue
Sol Rac1
Pellet Rac1
Tubulin
B
+SDS
Ctrl KD
Coro1C KD
Coro1C
Tubulin
Sol Rac1
Pellet Rac1
C
Coro1C
Actin
WT
Coro1C KD
D
RCC2 KD:
Merge
GFP-Coro1C
Actin
E
Membrane
Nuclear
Skeletal
RCC2: Ctrl KD Ctrl KD Ctrl KD
H/0: - + - + - + - + - + - +
RCC2
Rac1
Coro1C
F
Coro1C KD
Ctrl
Fluorescence Intensity
Time (sec)
G
Rate constant (s-1)
Ctrl
Coro1C KD oligo #2
Lateral
H
Rate constant (s-1)
**
ns
Ctrl
Ctrl
Coro1C KD
Lateral
Protrusion
I
D38 F37 T35 Y32
L
Bait
GST
GST-Rac1
RNAi
Ctrl
RCC2
Bound
Coro1C
RCC2
Input
Coro1C
RCC2
Tubulin
Coomassie
GST-Rac1
GST
M
Bait
GST
GST-Rac1
RNAi
Ctrl
Coro1C
Bound
Coro1C
RCC2
Input
Coro1C
RCC2
Tubulin
Coomassie
GST-Rac1
GST
J
GFP-Coro1C
WT R31E
Coro1C
RCC2
K
WT
R31E
R28D /2xKE
S P S P S P
GFP-Coro1C
Actin
N
Ctrl
RCC2 KD
Coro1C KD
Coro1C/ RCC2 KD
Coro1C/ RCC2 KD
GFP-Rac1
Actin
Anti Rac1
Actin
O
Number of protrusions per cell
Ctrl
RCC2 KD
Coro1C KD
Coro1C/ RCC2 KD

Figure S3.

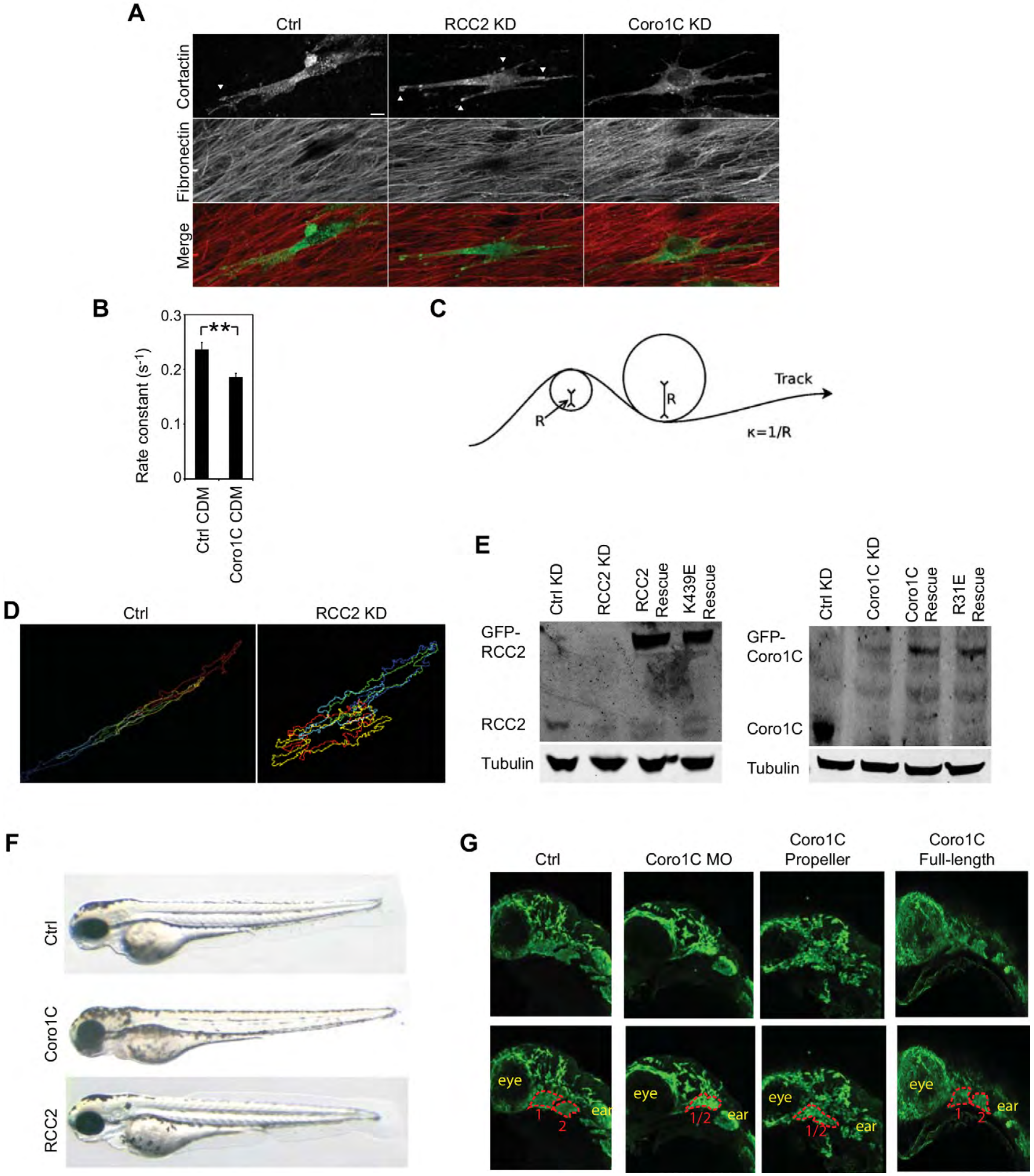
A
Ctrl
RCC2 KD
Coro1C KD
Cortactin
Fibronectin
Merge
B
Rate constant (s-1)
0.3
0.2
0.1
0
**
Ctrl CDM
Coro1C CDM
C
Track
R
R
κ=1/R
D
Ctrl
RCC2 KD
E
Ctrl KD
RCC2 KD
RCC2 Rescue
K439E Rescue
GFP-RCC2
RCC2
Tubulin
Ctrl KD
Coro1C KD
Coro1C Rescue
R31E Rescue
GFP-Coro1C
Coro1C
Tubulin
F
Ctrl
Coro1C
RCC2
G
Ctrl
Coro1C MO
Coro1C Propeller
Coro1C Full-length
eye
1
2
ear
eye
1/2
ear
eye
1/2
ear
eye
1
2
ear

Figure S4.

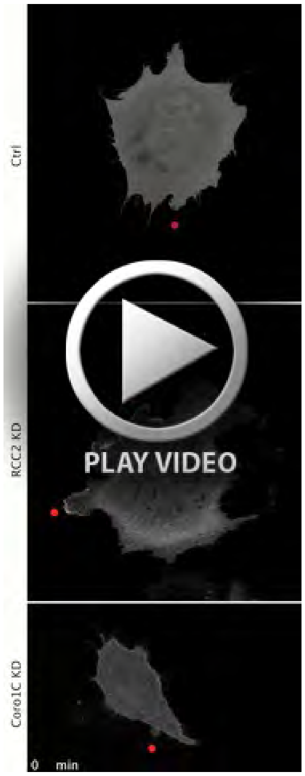

Movie 1.

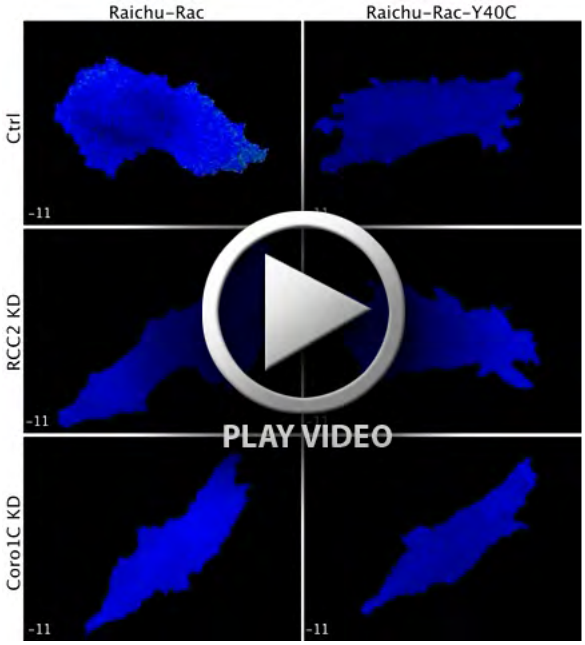

Movie 2.

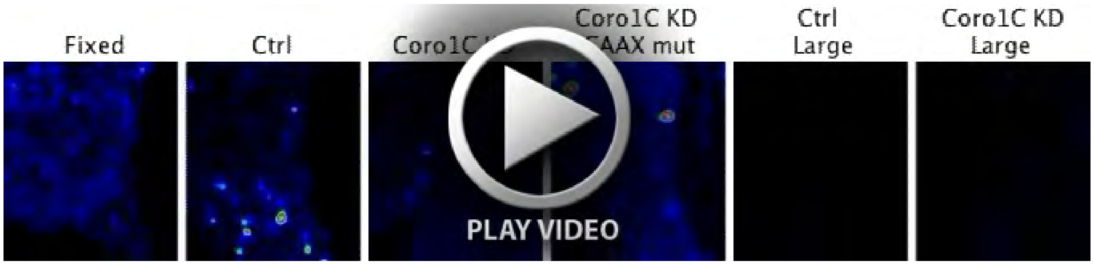

Movie 3.

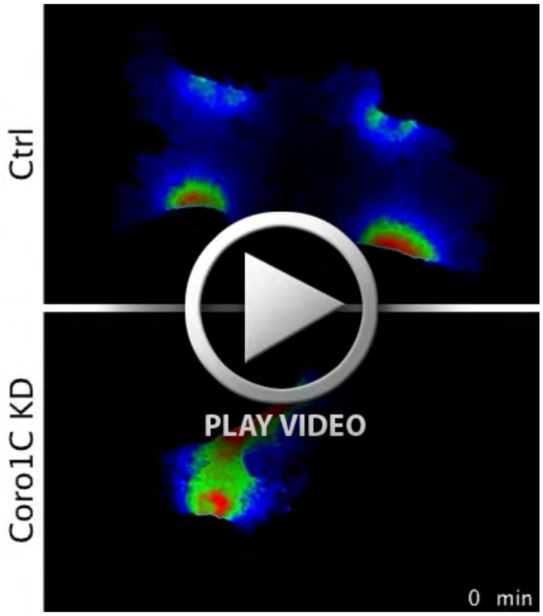

Movie 4.

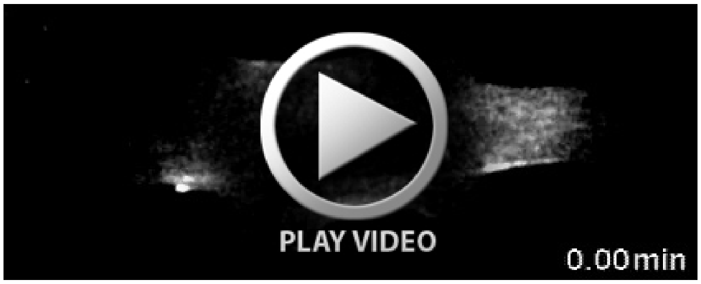

Movie 5.

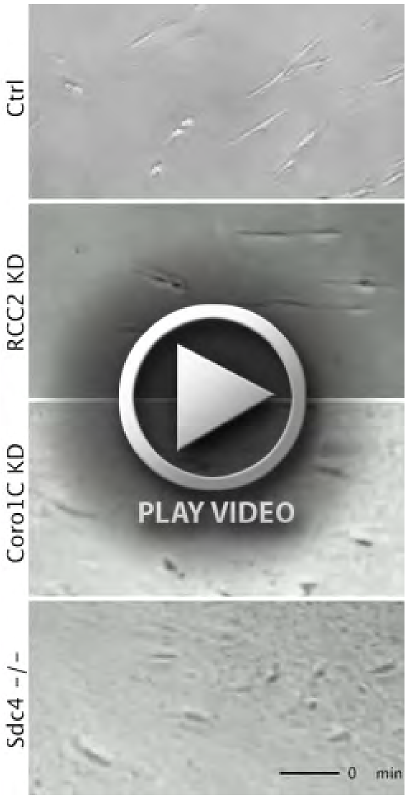

Movie 6.

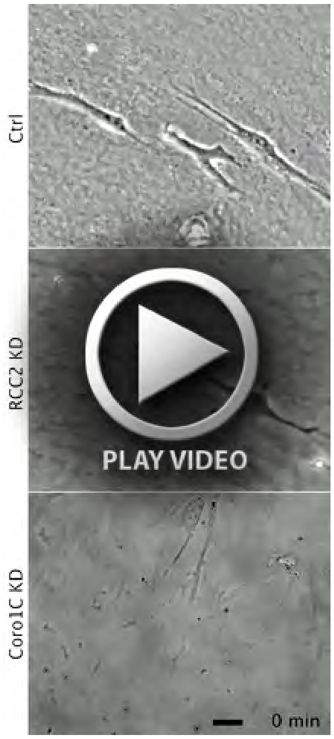

Movie 7.

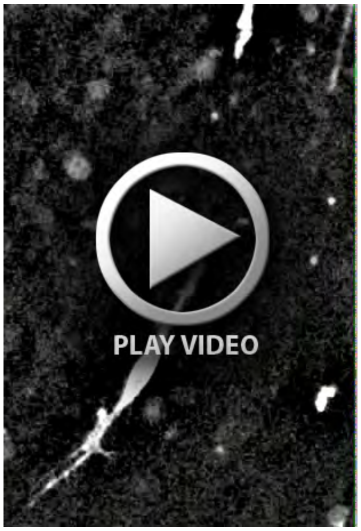

Movie 8.

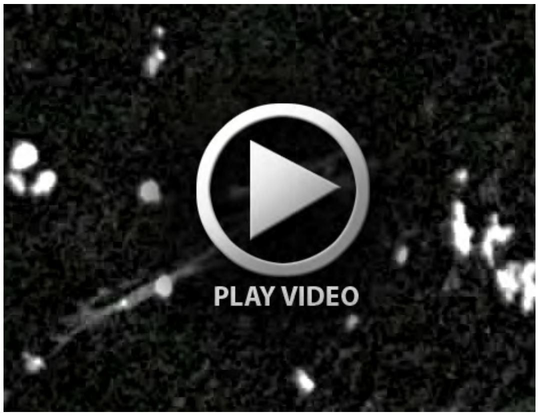

Movie 9.

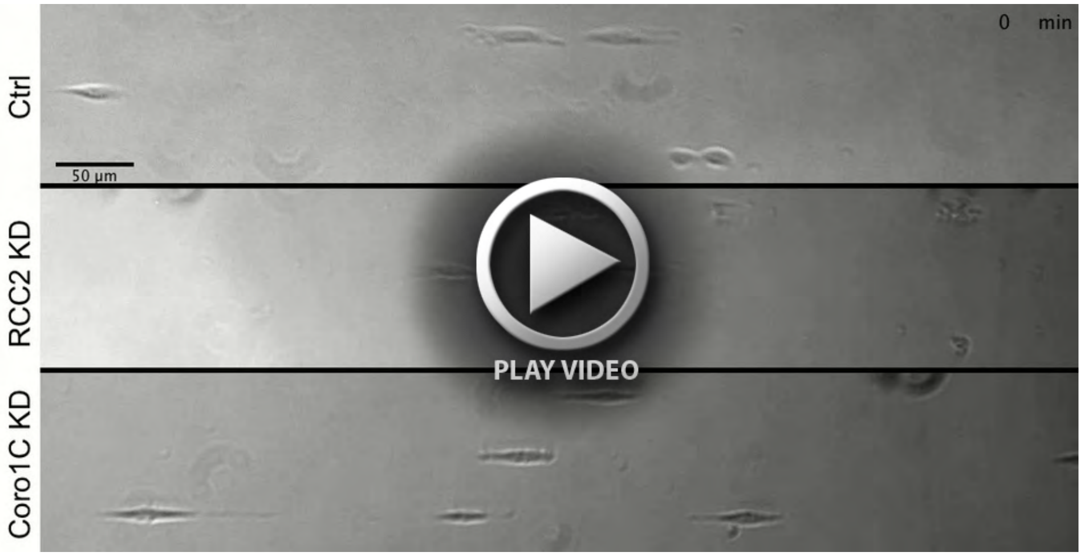

Movie 10.
